# Extracellular vesicles derived from mesenchymal stromal cells mitigate intestinal toxicity in a mouse model of acute radiation syndrome

**DOI:** 10.1186/s13287-020-01887-1

**Published:** 2020-08-27

**Authors:** Alison Accarie, Bruno l’Homme, Mohamed Amine Benadjaoud, Sai Kiang Lim, Chandan Guha, Marc Benderitter, Radia Tamarat, Alexandra Sémont

**Affiliations:** 1 Laboratory of Medical Radiobiology, Fontenay-aux-Roses, France; 2 Department of Research in Radiobiology and Regenerative Medicine, Institute for Radiological Protection and Nuclear Safety (IRSN), Fontenay-aux-Roses, France; 3 Laboratory of Accidental Radiobiology, Department of Research in Radiobiology and Regenerative Medicine, Institute for Radiological Protection and Nuclear Safety, Fontenay-aux-Roses, France; 4 Institute of Molecular and Cellular Biology, Agency for Science, Technology and Research, Singapore, Singapore; 5 Department of Radiation Oncology, Albert Einstein College of Medicine, Montefiore Medical Center, Bronx, NY USA

**Keywords:** Acute radiation syndrome, Gastrointestinal syndrome, Intestinal epithelial barrier, Medical countermeasures, Mesenchymal stromal cell-derived extracellular vesicles

## Abstract

**Background:**

Human exposure to high doses of radiation resulting in acute radiation syndrome and death can rapidly escalate to a mass casualty catastrophe in the event of nuclear accidents or terrorism. The primary reason is that there is presently no effective treatment option, especially for radiation-induced gastrointestinal syndrome. This syndrome results from disruption of mucosal barrier integrity leading to severe dehydration, blood loss, and sepsis. In this study, we tested whether extracellular vesicles derived from mesenchymal stromal cells (MSC) could reduce radiation-related mucosal barrier damage and reduce radiation-induced animal mortality.

**Methods:**

Human MSC-derived extracellular vesicles were intravenously administered to NUDE mice, 3, 24, and 48 h after lethal whole-body irradiation (10 Gy). Integrity of the small intestine epithelial barrier was assessed by morphologic analysis, immunostaining for tight junction protein (claudin-3), and in vivo permeability to 4 kDa FITC-labeled dextran. Renewal of the small intestinal epithelium was determined by quantifying epithelial cell apoptosis (TUNEL staining) and proliferation (Ki67 immunostaining). Statistical analyses were performed using one-way ANOVA followed by a Tukey test. Statistical analyses of mouse survival were performed using Kaplan-Meier and Cox methods.

**Results:**

We demonstrated that MSC-derived extracellular vesicle treatment reduced by 85% the instantaneous mortality risk in mice subjected to 10 Gy whole-body irradiation and so increased their survival time. This effect could be attributed to the efficacy of MSC-derived extracellular vesicles in reducing mucosal barrier disruption. We showed that the MSC-derived extracellular vesicles improved the renewal of the small intestinal epithelium by stimulating proliferation and inhibiting apoptosis of the epithelial crypt cells. The MSC-derived extracellular vesicles also reduced radiation-induced mucosal permeability as evidenced by the preservation of claudin-3 immunostaining at the tight junctions of the epithelium.

**Conclusions:**

MSC-derived extracellular vesicles promote epithelial repair and regeneration and preserve structural integrity of the intestinal epithelium in mice exposed to radiation-induced gastrointestinal toxicity. Our results suggest that the administration of MSC-derived extracellular vesicles could be an effective therapy for limiting acute radiation syndrome.

## Introduction

Accidental or intended catastrophic nuclear/radiological events represent a real threat of mass casualty catastrophe. In such events, the exposed victims would be considered “at increased risk” of developing exposure-related morbidity and/or mortality, called acute radiation syndrome (ARS). Whole-body irradiation (WBI) doses can be divided into potentially sublethal (≤ 2 Gy), lethal (between 2 and 10 Gy), and supralethal (≥ 10 Gy) [1–3]. For doses of between 2 and 6 Gy, the hematopoietic (HP) injury is expected to be the major contribution to victim mortality that occurs within weeks after exposure [2, 4, 5]. In this case, acute radiation exposure triggers the death of hematopoietic stem cells and progenitor cells leading to myelosuppression and increased susceptibility to infection, hemorrhage, and anemia [1–3, 6–8]. Following exposure to higher doses (between 6 and 10 Gy), victims develop irreversible HP and gastrointestinal (GI) injuries [1, 2, 9]. Radiation-induced GI syndrome is characterized by the death of GI stem/progenitor cells, the drastic functional dysregulation of the intestinal epithelium, and the loss of digestive barrier integrity [8, 10]. Victims suffer from abdominal pain, diarrhea, dehydration, intestinal bleeding, and sepsis, and mortality occurs within 2 weeks of exposure [3, 8]. Because the time window of opportunity for intervention is very short, between 24 and 48 h after exposure, most drugs under investigation are radioprotectors or radiomitigators. Nevertheless, in 2006, a European consensus was established for the treatment of accidental radiation-induced HP syndrome [11, 12]. This treatment involves the acute administration of a cytokine combination to stimulate residual hematopoiesis. In the case of irreversible medullar aplasia, bone marrow transplantation could be used. Although these treatments were efficacious for HP syndrome management, they were not able to rescue GI syndrome in experimental models [13, 14]. Consequently, GI syndrome remains intractable to clinical intervention and is lethal for patients exposed to high doses of radiation.

An innovative therapy using the administration of mesenchymal stromal cells (MSC) has been proposed for treating GI alterations in ARS syndromes [15, 16]. MSC have been reported to have pleiotropic properties and have been tested in more than 1000 clinical trials for the treatment of a wide range of diseases (http://www.clinicaltrials.gov), including ones affecting the bone marrow and GI tract. In 2006, it was proposed that MSC exert their therapeutic effects through secretion of bioactive factors [17, 18]. The action mechanisms of the paracrine physiological functions of MSC include one relating to their potential to release extracellular vehicles (EVs), namely MSC-derived small EVs and/or microvesicles [19–21]. The terms “MSC-derived small EVs” and “microvesicles” actually refer to two different EV types that differ through their biogenesis. MSC-derived small EVs are derived from endosomes [22, 23], while microvesicles are derived from the plasma membrane [24, 25]. Isolation of different EV types based on their biogenesis is currently not practical or possible because we lack specific biomarkers. The size and density of EVs are commonly used for their isolation, but these factors cannot distinguish among different EV types, whose biophysical parameters overlap [21]. Consequently, all MSC-derived EV preparations described to date are probably heterogeneous mixtures of different EV types whose biogenesis is unknown. In this study, we established that at least a fraction of the EVs in our preparation is derived from the endosome and so these EVs are MSC-derived small EVs [26]. However, at least two other EV types have been reported [27]. Since the MSC-derived EVs in our preparation are between 50 and 200 nm (in supplementary data 1), the term “MSC-derived EVs” in this paper is synonymous with “MSC-derived exosomes” as per recent recommendations [21, 28].

Extensive studies have shown the regenerative potential of MSC-derived exosomes in many injured organs such as the heart [29], the kidney [30], the liver [31], the skin [32], and also the intestine [33].

The benefit seems to be directly correlated to their ability to improve some processes taking place in each of the main stages of tissue repair. Indeed, MSC-derived exosomes have been described as attenuating inflammatory and oxidative stress responses, promoting angiogenesis and the re-epithelization process [30, 32, 34–37]. Similarly, the therapeutic benefits of MSC for radiation-induced intestinal toxicity might also be attributed to the release of EVs, particularly the MSC-derived exosomes, which were already implicated as the mediator of MSC protection in necrotizing enterocolitis of the intestine [33].

In this context, the aim of this study was to test the effect of MSC-derived EVs including exosomes on radiation-induced GI toxicity. We used a model consisting of mice subjected to lethal WBI in order to mimic the overlapping of multi-organ failure observed in ARS. To assess the therapeutic benefit of MSC-derived EVs, we chose a WBI dose of 10 Gy, inducing, as we reported, myelosuppression and high transient rupture of the intestinal barrier. We demonstrated that short-term MSC-derived EV therapy significantly delayed time to death in the WBI animals and this delay could be attributed to the maintenance of intestinal barrier integrity.

## Material and methods

All experiments were performed in compliance with the Guide for the Care and Use of Laboratory Animals, French regulations for animal experiments (Ministry of Agriculture Order No. B92-032-01, 2006), and European Directives (86/609/CEE) and approved by the Institute’s local ethics committee (permit number: D92-032-01, APAFIS#6503-2016082311257373v2).

### Experimental design

In a first batch of the experiment, 4 groups of mice were submitted to different doses (15, 13, 12, and 10 Gy) of whole-body irradiation (WBI) and one group was not irradiated (control group). Experiments were performed to assess the survival rate of mice after WBI (between 15 and 31 animals per group). Three days after WBI, intestinal barrier integrity was also studied by measuring in vivo intestinal permeability (between 10 and 20 animals per group) and assessing the histological data (between 12 and 18 animals per group). In a second batch of the experiment, 3 groups of mice were submitted to 10 Gy WBI and one group was not irradiated (control group). The irradiated mice were then treated with PBS, MSC, or MSC-derived EVs, and the non-irradiated mice received only PBS. All mice received the treatment intravenously, through the retro-orbital sinus, 6 h, 24 h, and 48 h after irradiation for MSC-derived EVs (200 μg for each injection) and 6 h after irradiation for MSC (5 million in one injection). Like the first batch of the experiment, 3 days after WBI, intestinal barrier integrity was also studied by measuring in vivo intestinal permeability (between 6 and 36 animals per group) and assessing the histological data (between 18 and 24 animals per group). Experiments to assess the survival rate of mice after 10 Gy WBI with or without therapy (MSC or MSC-derived EVs) were also performed (between 11 and 19 animals per group).

In a third batch of the experiment, 3 groups of mice were submitted to 10 Gy WBI and one group was not irradiated (control group). The mice received treatment as described above in the second batch of the experiment. Mice were then sacrificed 24 h after irradiation (at this time, the mice received a single injection of MSC or a single injection of MSC-derived EVs), 48 h after irradiation (at this time, the mice received a single injection of MSC or 2 injections of MSC-derived EVs), or 72 h after irradiation (at this time, the mice received a single injection of MSC or 3 injections of MSC-derived EVs) as shown in Fig. 3. In this experiment, the apoptosis and proliferation of epithelial crypt cells were assessed on small intestinal sections by TUNEL assay or immunostaining by KI67 antibody, respectively. Between 4 and 24 animals per group was used; the two first batch of experiments were realized three times, the third one only one time.

### Irradiation protocol

Male NUDE mice (Janvier SA, Le Genest St Isle, France) 6/8 weeks old were received and housed in a temperature-controlled room (21 ± 1 °C). They were allowed free access to water and fed standard pellets. The mice were anesthetized by a 2:1 (v/v) ketamine and xylazine mixture diluted in 0.9% NaCl and injected at 0.1 ml/g, and a single WBI dose was delivered by a medical accelerator (Alphée). Alphée is an accelerator-type radiation source (maximum energy 4 MeV with an average energy of about 1.5 MeV; 30 kA). The doses used were 15, 13, 12, and 10 Gy.

### Preparation and administration of human MSC

BM cells were obtained after receiving the informed consent of patients undergoing total hip replacement surgery, and these were used in accordance with the procedures approved by the human experimentation and ethics committees of Hospital St-Antoine (France). Between 10 and 20 ml of BM cells were harvested in α-MEM (Invitrogen, Cergy, France) supplemented with heparin. Total cells were then isolated from any bone fragments. Nucleated cells were plated at 50,000 cells/cm^2^ in α-MEM supplemented with 10% fetal calf serum (research grade FCS, Hyclone, Perbio, France), 1% l-glutamine, 1% penicillin streptomycin, and 1 ng/ml β-FGF (Sigma-Aldrich Chimie SARL, Lyon, France) as used in clinics [38]. Culture flasks were incubated at 37 °C with 5% CO_2_ in a humidified atmosphere. After 72 h, noncompliant cells were removed, and the medium was replaced twice a week until 90% confluence was reached. Samples of MSC from different donors were collected at passage 2 for transplantation.

At the time of infusion, the MSC were characterized by their expression of CD73 (SH3) and CD105 (SH2) and the lack of their expression of CD45 using FACS analysis and by their potential for osteogenic and adipogenic differentiation [39].

Five million of human MSC were intravenously administered in one injection 6 h after 10 Gy WBI. The controls received only the vehicle.

### Preparation and administration of MSC-derived EVs

MSC were obtained from immortalized E1-MYC 16.3 human embryonic stem cells. The deviation of the cell line was approved by the NUS Institution Review Board (IRB, title of research protocol: Derivation of Lineage-restricted cells from existing Human ES Cell Lines, the NUS-IRB approval number is NUS 188 and the NUS-IRB reference code is 05-090). All isolations and characterizations of MSC-derived EVs were performed as previously described [40, 41], but with some modifications. Briefly, immortalized E1-MYC 16.3 human embryonic stem cell-derived MSC were cultured in DMEM (GE Healthcare, USA) with 10% fetal bovine serum (FBS) (Thermofisher Scientific, Waltham, MA, USA). To obtain the EVs, 80% confluent cells were grown in a chemically defined medium for 3 days and the conditioned medium was harvested as previously described (see PMID 17565974). The conditioned medium was cleared of cell debris, fractionated, and concentrated 50× by tangential flow filtration using a membrane with a molecular weight cutoff (MWCO) of 100 kDa (Sartorius, Gottingen, Germany). Its EV yield was assayed by protein concentration using a NanoOrange Protein Quantification Kit (Thermofisher Scientific). Each batch of EV preparation was qualified for its particle size distribution (see below: nanoparticle tracking analysis in supplementary data 1) and presence of exosome-associated markers (see transmission electron microscopy in Supplementary data 1).

The MSC-derived EVs were lyophilized by Paracrine Therapeutics using a proprietary technique, stored at − 20 °C, and re-constituted at a concentration of 1 μg/μl with water for use. A total of 600 μg of MSC-derived EVs was intravenously administered in three 200-μg injections 6 h, 24 h, and 48 h after receiving 10 Gy WBI. The controls received only the vehicle.

#### Nanoparticle tracking analysis

The exosome was diluted 3000× with 0.22 μm filtered PBS. The exosome size distribution was then measured and analyzed by Zetaview® (Particle Metrix GmbH, Meerbusch, Germany) according to the manufacturer’s protocol.

#### Transmission on electron microscopy

Glow-discharged EM grids coated with Formvar/Carbon (EMS) were floated on a 20-μl drop of purified exosome fraction. The excess fluid was blotted away using filter paper, and the exosomes adhering to the grid surface were immune-labeled with mouse monoclonal anti-CD81 antibody (Santa Cruz Sc-7637) followed by goat anti-mouse secondary antibody coupled with 6 nm gold (EMS). Finally, the grids were fixed with 1% glutaraldehyde (EMS), washed, and embedded in a thin film of uranyl acetate-methylcellulose (a 4% uranyl acetate and 2% methylcellulose mixture in a 1:9 ratio) using the wire loop technique. Samples were analyzed under a JEOL transmission electron microscope (JEM-1010) operating at 80 kV and equipped with an SIA model 12C 4K CCD camera.

### Survival curve analysis

NUDE mice were exposed to 15 Gy, 13 Gy, 12 Gy, or 10 Gy lethal doses of WBI. The therapeutic effect of MSC-derived EVs was only assessed in NUDE mice subjected to 10 Gy WBI. Animal survival was monitored every 12 h.

### Histology methods

Formalin-fixed, paraffin-embedded small intestines were cut at 5 μm on a rotary microtome (Leica Microsystems AG, Wetzlar, Germany) and mounted on polysine slides. The sections were deparaffinized in xylene and rehydrated using ethanol baths and PBS. The hydrated sections were then stained with hematoxylin, eosin, and saffron (HES). The sections were studied for histological changes in the mucosa of the small intestine, and morphometric analyses were performed. We evaluated surviving crypts as a percentage of crypt containing 10 or more adjacent chromophilic cells and a lumen. We also assessed the villus height of the small intestine (in micrometer). For each section of the small intestine, between 30 and 100 measurements (depending on the severity of the lesion) were performed using image analysis software (Visiolab, Biocom, France).

### Immunohistochemistry

The hydrated sections were dipped into a permeabilization solution consisting of 0.1% Triton X-100 in PBS and rinsed in a distilled water bath for 5 min. Endogenous enzymes were then blocked using 3% hydrogen peroxide (H_2_O_2_) in methanol for 10 min and washed again in a 50-mM Tris buffer containing 9 g/l NaCl (TBS). To expose masked epitopes, the tissues were incubated for 30 min in 10 mmol/l buffered citrate (pH 6.0). Non-specific antibody binding was minimized by incubating the sections with a protein-block solution (DakoCytomation X0909, DakoCytomation, Courtaboeuf, France) for 30 min. The tissues were incubated in the presence of the primary antibody, Ki67 polyclonal rabbit anti-rat antibody (Abcam ab66155) or claudin-3 polyclonal rabbit anti-rat (Thermofisher, PA-16867) at a dilution of 1:1000 and 1:100, respectively, in Dako antibody diluent for 60 min at 37 °C in a humidified chamber. The sections were then rinsed in PBS buffer and then incubated with Envision kit anti-mouse HRP (K4002, DakoCytomation) for 30 min at RT. Staining was developed with Histogreen substrate (E109, Eurobio, Les Ulis, France). The sections were then counterstained with Nuclear Fast Red (H3403, VectorLabs, Burlingame, CA, USA), dehydrated, and mounted. Isotype control antibodies were used as negative controls. Proliferating cells were determined using image analysis software (Visiolab). A minimum of 30 crypts per section was measured. For each crypt, we evaluated the number of KI67-positive epithelial cells and expressed the results as the percentage of positive cells per crypt.

### TUNEL staining

The hydrated sections were incubated in a citrate buffer (pH 6) for 3 three 5-min cycles in a 600-W microwave oven, with 3 min between each followed by 5 min in tap water. The slides were saturated for 30 min with PBS BSA 1%, rinsed in PBS three times, and stained with the In-Situ Cell Death Detection Kit (Roche Diagnostics) according to the manufacturer’s guidelines. They were then mounted with DAPI (Vector Laboratories, USA). Apoptotic cells were determined using image analysis software (Visiolab). A minimum of 30 crypts per section was measured. For each crypt, we evaluated the number of TUNEL-positive epithelial cells and expressed the result as the percentage of positive cells per crypt.

### In vivo intestinal permeability assay

In vivo intestinal permeability was assessed using fluorescein dextran (FITC-Dextran 4, Sigma-Aldrich) as previously described [42]. The mice were orally gavaged with 0.75 mg/g body weight of 4 kDa FITC-labeled dextran, and blood samples were obtained from the retro-orbital venous plexus 5 h after this administration. Blood samples were centrifuged for 10 min at 5000 rpm, and plasma was taken and frozen at − 20 °C and analyzed the following day. Intestinal permeability to 4 kDa FITC-labeled dextran was determined by measuring the fluorescence intensity in plasma at 485 nm/525 nm using an automatic Infinite M200 microplate reader (Tecan, Lyon, France).

### Statistical analysis

Data are given as the mean ± SEM (standard error of the mean). The results were compared between groups by one-way ANOVA followed by a Tukey test using GraphPad 7.0 software (GraphPad, San Diego, CA). The results were also compared between groups using normal or Poisson regression models according to the nature of the parameter of interest, continuous response (villus height and intestinal permeability) or count data (apoptosis and proliferation), respectively.

The mouse survival curves were calculated by using the Kaplan-Meier method, and the *p* value was determined by a log-rank test possibly adjusted for multiple comparisons. The Cox survival model was used to assess the association between the MSC-derived EV therapy and the risk of death [43]. The coefficients in a Cox regression relate to hazard, which quantifies an increase in the risk or a protective effect depending on the sign of the fitted coefficients (positive or negative, respectively). The significance analyses were set at ***p ≤ 0.0001, **p ≤ 0.001, *p ≤ 0.05 vs *the* non-irradiated group; at ^§§§^*p* ≤ 0.0001, ^§§^*p* ≤ 0.001, ^§^*p* ≤ 0.05 vs the 10-Gy WBI group; and at ^###^*p* ≤ 0.0001, ^##^*p* ≤ 0.001, ^#^*p* ≤ 0.05 irradiated mice treated with MSC vs irradiated mice treated with MSC-derived EVs. The regression and survival analyses were conducted using MATLAB version 8.2.0.701 (R2013b), and the graphical representations were generated using GraphPad 7.0 software (GraphPad, San Diego, CA).

## Results

### Characterization of the NUDE mouse model: WBI-induced severe hematopoietic injury is associated with dose-dependent small intestine damage

**Fig. 1:**
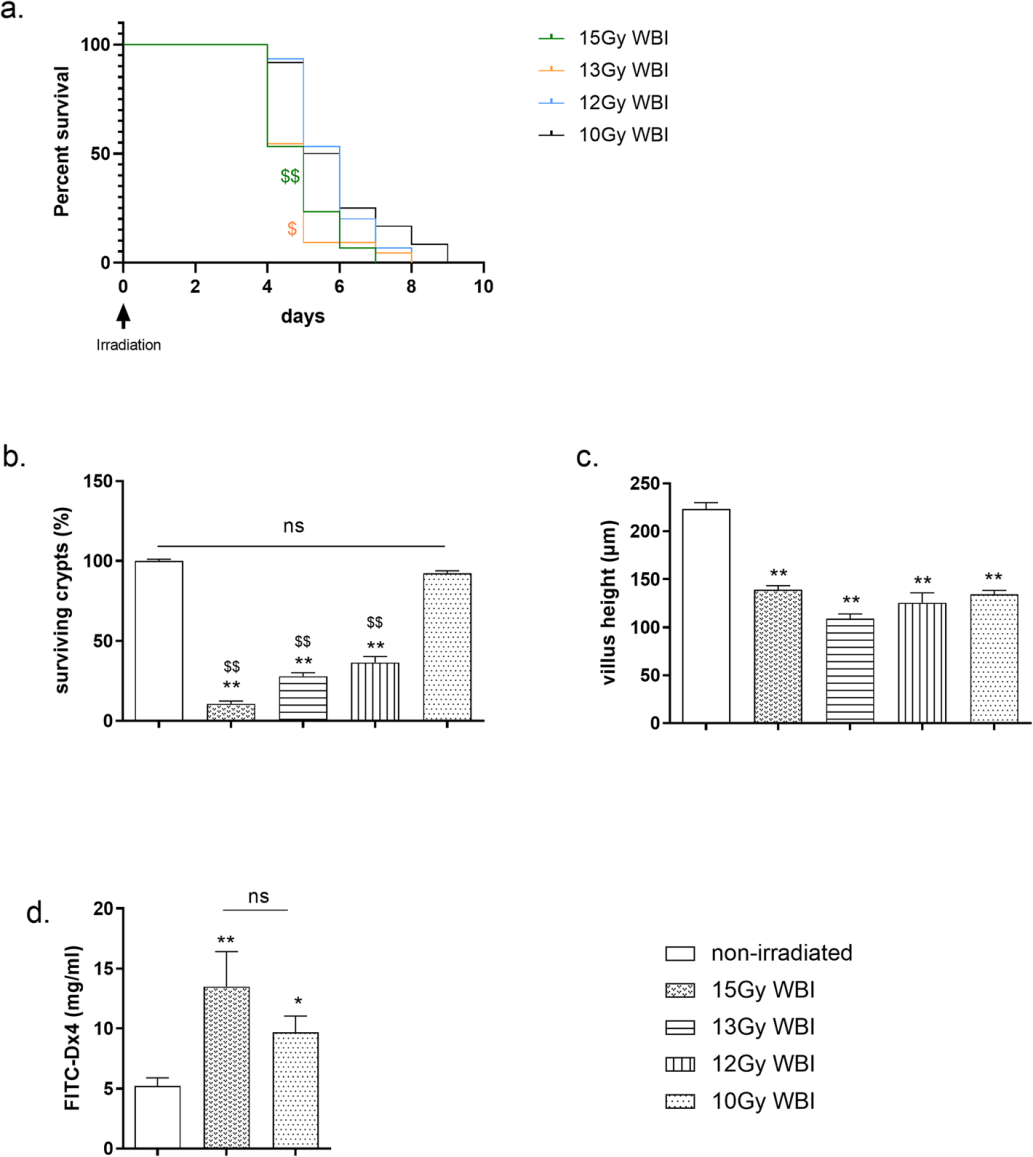
Characterization of a lethal WBI model in NUDE mice. **a**–**c** NUDE mice were subjected to 15 Gy, 13 Gy, 12 Gy, and 10 Gy lethal doses of WBI. **a** The survival rate of the mice was evaluated. Each value represents the cumulative data of 3 independent experiments (*N* = 3, 31 mice in the 15-Gy WBI group, 21 mice in the 13-Gy group WBI, 15 mice in the 12-Gy WBI group, and 23 mice in the 10-Gy WBI group). Mouse survival curves were calculated using the Kaplan-Meier method, and the *p* value was determined by the log-rank test and Cox model, ^$^*p* ≤ 0.05 and ^$$^*p* ≤ 0.001 compared to the 10-Gy WBI group. **b**, **c** Three days after WBI, small intestinal sections were stained with hematoxylin-eosin-safran (HES). Morphometric analysis was performed: **b** quantitative assessment of the small intestinal crypt viability to determine the percentage of surviving crypts containing 10 or more adjacent chromophilic cells and a lumen, and **c** villus height (μm). Each value represents the average of 30–100 independent measurements (where the decreasing doses we observed in each section increased the number of measurable crypts and villi) coming from 3 independently repeated experiments (*N* = 3, 4 to 6 animals per group and experiment). **p ≤ 0.001 compared with the non-irradiated group, ^$$^*p* ≤ 0.001 compared with the 10-Gy WBI group. **d** Nude mice received 15 Gy or 10 Gy WBI. At 3 days, non-irradiated and irradiated mice were orally gavaged with 75 mg/ml of 4 kDa FITC-labeled dextran, and 5 h later, blood samples were collected. Small intestinal permeability was determined by a plasmatic dosage of 4 kDa FITC-labeled dextran (mg/ml). Each value represents the average value coming from 2 independently repeated experiments (*N* = 2, 5 to 10 animals per group and experiment). *p ≤ 0.05, **p ≤ 0.001 compared with the non-irradiated group

In this part of the study using a severe injury-induced death model, we determined the limiting doses of WBI that induce rupture of the gut barrier. Mice were subjected to decreasing lethal WBI doses of between 15 and 10 Gy. Reduction in WBI doses was associated with an increase in mouse survival time (Fig. 1a). The maximum mouse survival time was 7 days for 15 Gy, 8 days for 13 or 12 Gy, and 9 days for 10 Gy. Moreover, the risk of instantaneous death after 10 Gy was reduced by a factor of 3.75 compared with 15 Gy (*p* ≤ 0.001, Cox model) and by 2.75 compared with 13 Gy (*p* ≤ 0.05, Cox model). No difference in the risk of instantaneous death was observed between mice subjected to 12 or 10 Gy. Reducing the doses also led to less weight loss, 5 days after irradiation (in supplementary data 2, *p* ≤ 0.001 for all tested doses vs non-irradiated mice). There was similar weight loss in mice receiving 15 or 13 Gy (in supplementary data 2, 28 and 30% loss vs non-irradiated mice) and in mice receiving 12 or 10 Gy (in supplementary data 2, 20% loss vs non-irradiated mice). However, we observed significant differences in weight loss between 15 or 13 Gy vs 12 or 10 Gy (in supplementary data 2, *p* ≤ 0.001 for 15 Gy vs 12 and 10 Gy and *p* ≤ 0.05 and *p* ≤ 0.001 for 13 Gy vs 12 and 10 Gy, respectively). For all WBI doses tested, histological analysis showed similar severe damage in bone marrow that was indicative of myelosuppression (in supplementary data 3 and 4a). Allogenic transplantation of total bone marrow (BM) in irradiated mice avoids myelosupression and is usually used as a gold standard test to characterize the WBI dose needed to cause intestinal injury-dependent death [44]. In previous personal experiments, allogenic transplantation of total BM in NUDE mice (5 million cells per mouse) was performed intravenously by retro-orbital injection, 2 h after a range of WBI doses similar to those used in this study (in supplementary data 4). Although the transplantation increased the number of hematopoietic sites in the BM, this treatment did not prevent mouse death (in supplementary data 4). This test supports the thesis that WBI-induced death seems to be mainly triggered by intestinal injury following such WBI doses. Consequently, we measured crypt cell viability as the first criterion of intestinal injury. Three days after the WBI, we showed a dose-dependent effect on the percentage of surviving crypts in the small intestine. Only 10% of the crypts were viable after the highest dose (15 Gy), suggesting that the epithelium is unable to repair and regenerate itself (Fig. 1b, *p* ≤ 0.001 vs non-irradiated mice). In support of this suggestion, we also reported the severe structural epithelial alterations, shown in Fig. 1c and supplementary data 5a respectively affecting the villus atrophy area and crypt depth (villus height reduction, *p* ≤ 0.001 vs non-irradiated mice and crypt depth tendency vs non-irradiated mice), and in supplementary data 5b, regarding the ulceration areas (confirmed by the crypt/villus density reduction, *p* ≤ 0.001 vs non-irradiated mice). This was concomitant with the 2.6-fold increase in intestinal permeability (Fig. 1d, 5.2 ± 0.7 in non-irradiated mice vs 13.5 ± 2.9 in 15 Gy irradiated mice, *p* < 0.001 vs non-irradiated mice). Fifteen grays of WBI induced severe and irreversible small intestinal disorders and death at between 4 and 7 days.

At the lowest, 10 Gy dose, WBI did not affect crypt viability (Fig. 1b). Nevertheless, we observed the transient (only at 3 days, data not shown at 5 days) disruption of the intestinal barrier as shown by the significant villus atrophy seen in Fig. 1c (223.2 ± 6.6 μm in non-irradiated mice vs 134.8 ± 3.9 μm in 10 Gy irradiated mice, *p* < 0.001 vs non-irradiated) and the 1.9-fold increase in intestinal permeability shown in Fig. 1d (5.2 ± 0.7 mg/ml 4 kDa FITC-labeled dextran in non-irradiated mice vs 9.7 ± 1.6 mg/ml 4 kDa FITC-labeled dextran in 10 Gy irradiated mice, *p* < 0.05 vs non-irradiated mice). A 10-Gy WBI dose induced short-term small intestinal disorders that although reversible led to death at between 4 and 9 days.

### Significant therapeutic benefit of MSC-derived EVs in irradiated NUDE mice developing overlapping hematopoietic and intestinal injuries

Based on the dose-effect observations, we opted for a 10-Gy WBI dose for the rest of the experiments. This dose of irradiation generated ARS with severe small intestinal injury, but preserved sufficient surviving crypts to support therapeutic intervention to repair and regenerate the crypts, and it provides a first proof of concept for the therapeutic efficacy of MSC-derived EVs in ARS management.

Based on assays evaluating the dose-dependent effects of MSC-derived EVs (shown in supplementary data 6), we chose to administer a total of 600 μg of MSC-derived EVs in three 200-μg injections 6 h, 24 h, and 48 h after WBI.

#### MSC-derived EVs extend the life of irradiated NUDE mice

**Fig. 2:**
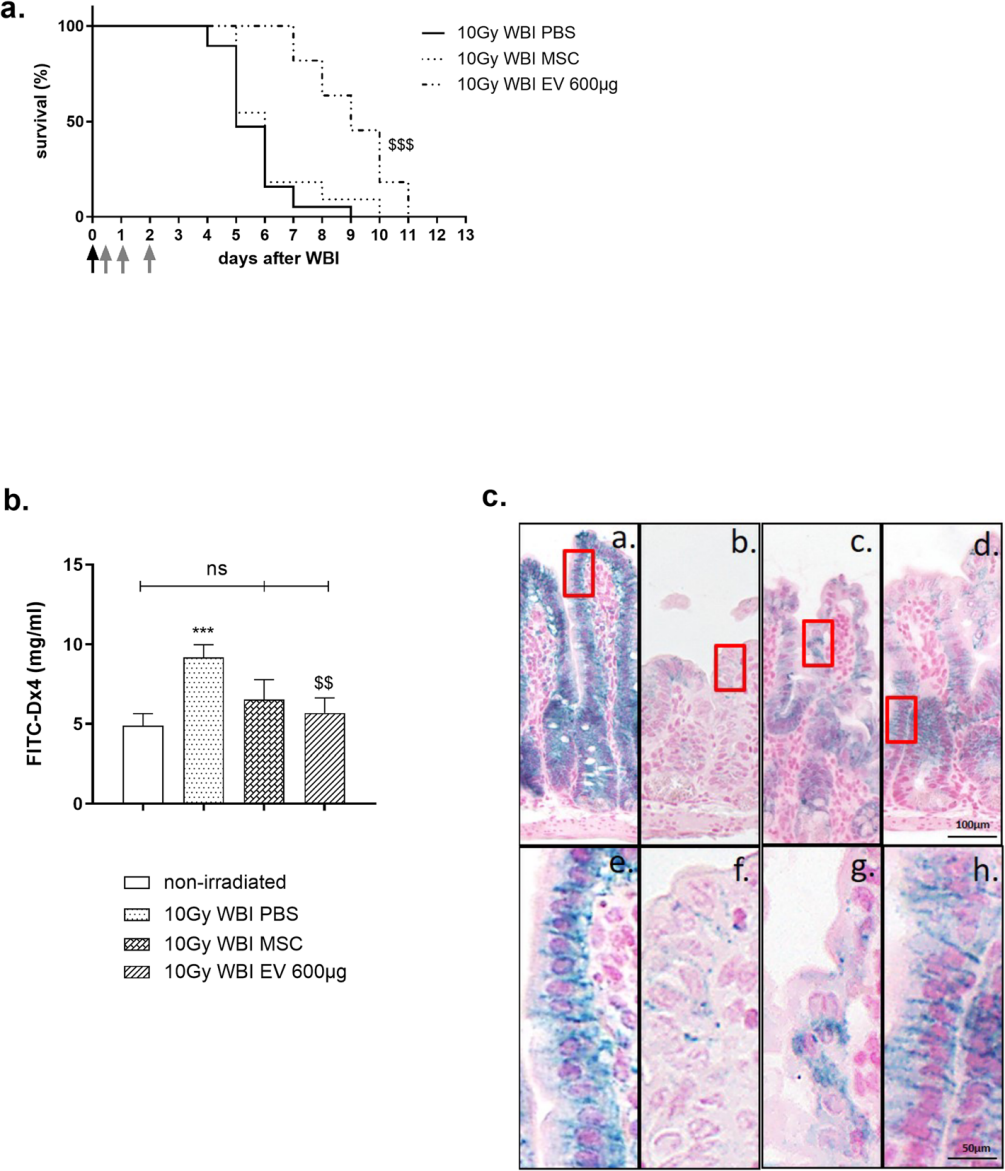
MSC-derived EVs extend the life and reduced intestinal permeability of NUDE mice subjected to 10 Gy WBI. **a**–**c** Nude mice received 10 Gy WBI (**a**) black arrow. Mice were randomly assigned to three different groups. Control mice received the vehicle (first group). MSC were intravenously administered in a single injection of 5 million cells, 6 h after WBI (second group (**a**), only the first gray arrow). A total of 600 μg of MSC-derived EVs was intravenously administered in three 200-μg injections 6 h, 24 h, and 48 h after WBI (third group (**a**), the three gray arrows). **a** The survival rate of mice was evaluated. Each value results from one representative experiment (*N* = 1, 19 mice in the 10-Gy WBI group treated with PBS, 11 mice in the 10-Gy WBI group treated with MSC, and 11 mice in the 10-Gy WBI group treated with MSC-derived EVs). The mouse survival curves were calculated using the Kaplan-Meier method, and the *p* value was determined by the log-rank test eventually adjusted for multiple comparisons. ^$$$^*p* ≤ 0.0001 compared with the 10-Gy WBI group. The survival Cox model was used in the assessment of the association between MSC-derived EV treatment and the instantaneous risk of death. **b** Three days after WBI, all mice were orally gavaged with 75 mg/ml of 4 kDa FITC-labeled dextran, and 5 h later, blood samples were collected. The small intestinal permeability was determined by a plasmatic dosage of 4 kDa FITC-labeled dextran (mg/ml). Each value represents the average values coming from 3 independently repeated experiments (*N* = 3, 2 to 12 animals per group and experiment), ***p ≤ 0.0001 compared with the non-irradiated group, ^$$^*p* ≤ 0.001 compared with the 10-Gy WBI group. **c** Representative pictures of claudin-3 immunostaining in the small intestine of non-irradiated NUDE mice (1 and 5), 3 days after 10 Gy WBI (2 and 6), 3 days after 10 Gy WBI and treatment with 5.10^6^ hMSC (3 and 7), and 3 days after 10 Gy WBI and treatment with MSC-derived EVs. Scale bar 100 μm for (1–4) and 50 μm for (5–8)

In our model, MSC-derived EVs, but not MSC, induced significant therapeutic efficacy as shown by their ability to delay 10 Gy WBI-induced death (Fig. 2a, log-rank test *p* < 0.0001). Five days after WBI when 50% of the mice had mostly died from intestinal toxicity, 100% of the mice treated with MSC-derived EVs were still alive. MSC-derived EVs delayed death at the lethal dose of 50% (LD50) in mice by 3.5 days compared with the untreated WBI mice. Consistent with these observations, the risk of instantaneous death induced by 10 Gy WBI was reduced by a statistically significant 85% (Cox model hazard ratio = 0.15, *p* ≤ 0.0001)*.*

#### MSC-derived EVs reduce gut barrier dysfunction after WBI in NUDE mice

Measurements of intestinal permeability and immunostaining of some transmembrane proteins of tight junctions were used as indexes of gut barrier function [45, 46]. We demonstrated that 10 Gy WBI induced a transient 1.8-fold enhancement of gut permeability (Fig. 2b, *p* ≤ 0.0001). MSC-derived EVs produced a more pronounced and significant effect than MSC in reducing radiation-induced hyper-permeability. In fact, 3 days after WBI, MSC-derived EVs were able to prevent radiation-induced increased gut permeability as shown in Fig. 2b, with significant difference in plasma fluorescein dextran concentrations between irradiated and EV-treated mice and irradiated mice but not in non-irradiated mice (Fig. 2b, 5.7 ± 1.0 mg/ml vs 9.1 ± 0.8 mg/ml; *p* < 0.05 and 4.9 ± 0.8 mg/ml, respectively). The MSC-treated group showed a numerically decreased but not significant concentration of fluorescein dextran compared to the irradiated mice (Fig. 2b). Claudin-3 plays an important role in safeguarding gut barrier function [46, 47]. It is a transmembrane protein of the tight junctions mainly localized at the intercellular junctions of the gut epithelium (Fig. 2c (a, e)) [46, 47]. Among the other proteins of tight junctions such as ZO-1 and occludin (data not shown), only claudin-3 immunostaining in the small intestine drastically decreased 3 days after 10 Gy WBI (Fig. 2c (b, f)). The lower junctional claudin-3 level after WBI could partly explain the increase in intestinal permeability. Treatment with MSC or MSC-derived EVs maintained a significant level of claudin-3 immunostaining despite a reduction in expression compared with the basal level. After MSC was administered, claudin-3 immunostaining was mostly observed in the cytoplasm (Fig. 2c (c, g)). In contrast, after MSC-derived EVs were administered, the claudin-3 remained localized at the membrane junction, meaning that the tight junctions had been preserved (Fig. 2c (d, h)). In conclusion, MSC-derived EVs were better able than MSC to limit WBI-induced disruption of the small intestinal barrier.

#### MSC-derived EVs stimulate the renewal of the small intestine and improve the regenerative process in irradiated NUDE mice

We first analyzed the time-dependent effect (1, 2, and 3 days after WBI) of MSC or MSC-derived EVs on the level of both apoptotic and proliferating cells in the small intestinal crypts as an index of the regenerative capacity of the epithelium. Villus height (the epithelium’s functional compartment) and crypt depth (the epithelium’s stem/progenitor compartment), as indexes of epithelial thickness and therefore of structural integrity, were assessed to demonstrate treatment efficacy in epithelium rescue.

#### Apoptosis analysis (Fig. 3b, c)

The physiological level of the apoptotic cells per crypt assessed by TUNEL assay in control mice was very low. The average value quantified was 1.5 ± 0.2% apoptotic cells per crypt. One day after 10 Gy WBI, we observed a significant 9-fold increase in apoptotic cells compared with the basal level (*p* ≤ 0.0001). This increase was lower on days 2 and 3, but was still significant being 5- and 3-fold times higher than the basal level, respectively (both *p* ≤ 0.0001). Administration of MSC or MSC-derived EVs significantly reduced the radiation-induced apoptosis of epithelial crypt cells 1 and 2 days after exposure (day 1—6.2 ± 0.8% for MSC-treated mice and 3.6 ± 0.6% for EV-treated mice, vs 13.2 ± 1.5% in irradiated mice, *p* ≤ 0.0001; day 2—3.8 ± 0.6% for MSC-treated mice and 2.2 ± 0.5% for EV-treated mice, vs 8.4 ± 1% in irradiated mice, *p* ≤ 0.0001). At 1 day, this effect was more pronounced after MSC-derived EV therapy in irradiated mice (*p* ≤ 0.001 vs MSC-treated mice). At 2 days, MSC-derived EVs, but not MSC, provided a prompt return to the basal level of epithelial apoptotic cells (2.2 ± 0.5% in irradiated and EV-treated mice vs 1.5 ± 0.2% in non-irradiated mice, *p* = 0.23). After MSC therapy, crypt apoptosis returned to the basal level in 3 days, 1 day later than the effect produced by MSC-derived EVs.

**Fig. 3:**
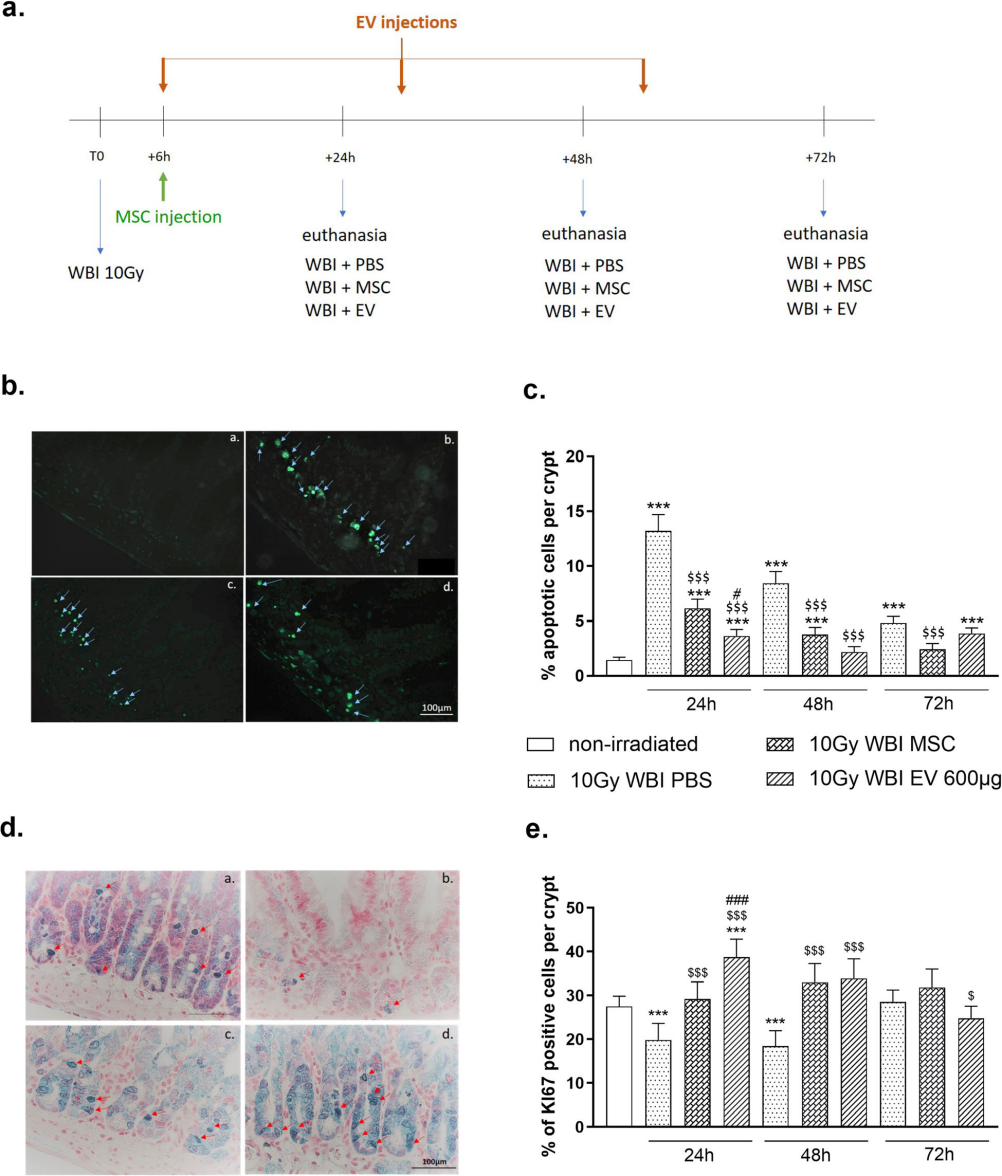
MSC-derived EVs reduce 10 Gy WBI-induced epithelial apoptosis and surge their proliferation. Nude mice received 10 Gy WBI. **a** Schema of the experimental protocol. Irradiated mice were then randomly assigned to three different groups. The first group received one intravenous injection of 200 μg MSC-derived EVs 6 h after WBI and were then sacrificed 24 h post-irradiation. The second group received two injections of 200 μg each of MSC-derived EVs 6 h and 24 h after WBI and were then sacrificed 48 h post-irradiation. The third group received three injections of 200 μg each of MSC-derived EVs 6 h, 24 h, and 48 h after WBI and were then sacrificed 72 h post-irradiation. The fourth, fifth, and seventh groups received 5.10^6^ MSC 6 h after WBI and were then sacrificed 6 h, 24 h, or 72 h post-irradiation, respectively. **b**–**e** Crypt cell apoptosis (**b**) and proliferation (**d**) were respectively assessed by TUNEL assay and KI67 immunostaining performed on small intestinal sections. Representative pictures from non-irradiated mice (**b** (a), **d** (a)), mice subjected to 10 Gy WBI and treated with PBS (**b** (b), **d** (b)), mice subjected to 10 Gy WBI and treated with MSC (**b** (c), **d** (c)), and mice subjected to 10 Gy WBI and treated by MSC-derived EVs (**b** (d), **d** (d)). Arrows show cells with the highest positive staining. Quantification of **c** crypt cell apoptosis and **d** crypt cell proliferation in non-irradiated NUDE mice (12 and 24 animals, respectively), 24 h, 48 h, or 72 h after 10 Gy WBI with PBS treatment (5 and 4 mice at 24 h, 6 and 5 mice at 48 h, and 6 and 17 mice at 72 h, respectively); 24 h, 48 h, or 72 h after 10 Gy WBI with 5.10^6^ MSC treatment (6 and 4 mice at 24 h, 6 and 4 mice at 48 h, and 3 and 3 mice at 72 h, respectively); or 24 h, 48 h, or 72 h after 10 Gy WBI with MSC-derived EVs (7 and 6 mice at 24 h, 6 and 5 mice at 48 h, and 6 and 7 mice at 72 h, respectively). Each value represents the average of 30 independent measurements per crypt and is expressed as a percentage of the non-irradiated value, *N* = 1. ***p ≤ 0.0001 compared with the non-irradiated group; ^$^*p* ≤ 0.05, ^$$$^*p* ≤ 0.0001 in comparison between the 10-Gy WBI group treated with MSC-derived EVs and the 10-Gy WBI group treated with PBS; ^#^*p* < 0.05, ^###^*p* < 0.0001 in comparison between the 10-Gy WBI group treated with MSC-derived EVs and the 10-Gy WBI group treated with MSC

#### Crypt cell proliferation analysis (Fig. 3d, e)

The estimated basal proliferation (proportion of Ki67-positive cells among the analyzed ones) was 27.9 ± 2.4% in non-irradiated mice. One day and 2 days after WBI, this basal proliferation fell by approximately a third to 19.8 ± 3.7% (*p* ≤ 0.0001 vs non-irradiated mice) and 18.4 ± 3.6% (*p* ≤ 0.0001 vs non-irradiated mice), respectively. Three days after WBI, the proliferating crypt cells returned to the basal level (28.6 ± 2.71% in irradiated mice vs 27.9 ± 2.4% in non-irradiated mice, *p* = 0.49). These results suggested that 3 days after WBI, the small intestine began to heal through crypt cell proliferation.

MSC were able to prevent the radiation-induced reduction of proliferating crypt cells at 1 day (29.24 ± 3.8% in irradiated and MSC-treated mice vs 19.8 ± 3.7% in irradiated mice, *p* < 0.0001) and contributed to an increase in proliferating cells compared with the irradiated group until 2 days after WBI (33.0 ± 4.2% in irradiated and MSC-treated mice vs 18.42 ± 3.57% in irradiated mice, *p* < 0.0001) but also trend to increase compared with the non-irradiated mice (33.0 ± 4.2% in irradiated and MSC-treated mice vs 27.5 ± 2.4% in controls, *p* = 0.08). At 3 days, the MSC-treated group was comparable to the non-irradiated group (31.8 ± 4.15% vs 27.9 ± 2.36%, *p* = 0.22). Following treatment with MSC-derived EVs, this process had already begun at 1 day with a 1.4-fold increase in proliferating cells compared with the basal level (38.7 ± 4.1% in irradiated and EV-treated mice vs 27.9% in non-irradiated mice, *p* = 0.0004) and was numerically maintained at 2 days (33.8 ± 4.5% in irradiated and EV-treated mice vs 27.9% in non-irradiated mice, *p* = 0.07). The MSC-derived EV-induced proliferation process returned to the basal level 3 days after WBI (24.8 ± 2.7% in irradiated and EV-treated mice vs 27.9% in control mice, *p* = 0.13). Consequently, MSC-derived EVs induce a rapid (starting earlier than after MSC treatment) yet transient acceleration in crypt cell proliferation, and possibly promote epithelial renewal.

#### Structural analysis

**Fig. 4:**
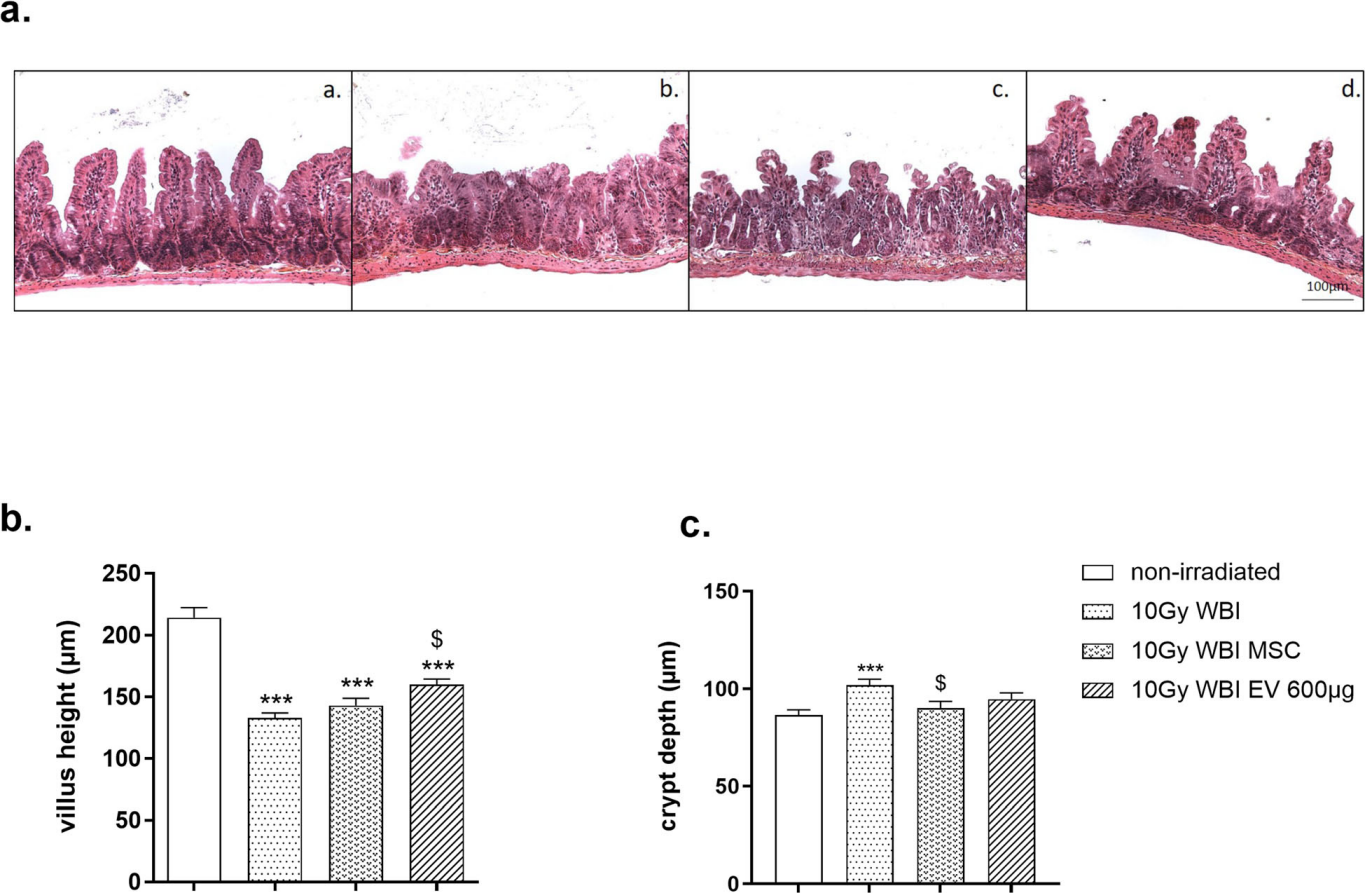
EVs minimize 10 Gy WBI-induced alteration of the small intestinal structure at 3 days. Nude mice received 10 Gy WBI and were sacrificed at 3 days. A total of 600 μg of MSC-derived EVs was intravenously administered in three 200-μg injections 6 h, 24 h, and 48 h after WBI. MSC were intravenously administered with one injection of 5 million cells, 6 h after WBI. Control mice received the vehicle. **a** Small intestinal sections were stained with hematoxylin-eosin-safran (HES). Representative pictures of small intestinal slices from non-irradiated mice (**a** (a)), mice subjected to 10 Gy WBI and treated with PBS (**a** (b)), mice subjected to 10 Gy WBI and treated with MSC (**a** (c)), and mice subjected to 10 Gy WBI and treated by MSC-derived EVs (**a** (d)). Scale bar 100 μm. Quantitative analysis of **b** the villus height of the small intestine (μm) and **c** the crypt depth of the small intestine (μm). Each value represents the average of 20–30 independent measurements per animal coming from 3 independently repeated experiments (*N* = 3, 6 to 8 animals per group and experiment). ***p < 0.0001 compared with the non-irradiated group, ^$^*p* < 0.05 compared with the 10-Gy group

As shown in Fig. 4, the villus height measured in control mice was 213.9 ± 8.3 μm (Fig. 4b) and the crypt depth was 86.5 ± 2.6 μm (Fig. 4c). A 10-Gy WBI dose led to partial atrophy of the functional epithelium at 3 days, corresponding to a significant reduction of the villus height to 132.8 ± 4.1 μm (*p* ≤ 0.0001 vs non-irradiated mice). In contrast, the crypt depth rose significantly to 101.9 ± 3 μm (*p* ≤ 0.0001 vs non-irradiated mice), contributing to mucosal healing beginning 3 days after WBI. MSC administration in irradiated mice induced a non-significant 7.5% increase in the villus height at 3 days compared with the average value of irradiated mice (142.9 ± 5.9 μm in irradiated mice treated by MSC vs 132.9 ± 4.1 μm in irradiated mice). At the same time, villus height in irradiated mice after MSC-derived EVs were administered was 159.8 ± 4.6 μm, corresponding to a significant 20.0% rise compared with the average value obtained in irradiated mice (*p* = 0.016). Crypt depth was decreased following both treatments (11% for MSC-treated mice and 7% for MSC-derived EVs) but only become significant for MSC-treated mice (*p* < 0.05; 90.0 ± 3.4 μm in irradiated mice treated by MSC vs 101.9 ± 3.0 μm in irradiated mice). No significant crypt density change in the small intestine was seen after WBI with or without MSC or MSC-derived EV treatments (data not shown). This part of the seems to show that MSC-derived EVs acted more rapidly and were more effective than MSC in preventing the loss of structural mass in the small intestine, possibly by increasing cellular proliferation and reducing apoptosis.

## Discussion

The manifestation of ARS is multifactorial and involves several overlapping complex mechanisms. Consequently, the development of therapeutics for this syndrome is challenging. We hypothesized that MSC-derived EVs with their pleiotropic potency would also be highly effective in the management of ARS. In accordance with this hypothesis, we provided the first proof of concept that MSC-derived EVs are a promising therapeutic for mitigation of radiation-induced toxicity.

Here, we report that the administration of MSC-derived EVs including exosomes acts through several processes to increase the survival time of mice that had developed multi-organ injuries and being expose to 10 Gy WBI. As GI syndrome is the critical limitation in combatting ARS effectively, we chose to focus on assessing the efficacy of MSC-derived EVs helping to restore GI mucosal barrier integrity following irradiation. Barrier impairment could lead to life-threatening sepsis. As sepsis is a major cause of death in GI syndrome [2] and the GI mucosal barrier is highly sensitive to radiation, preservation of this barrier is a major therapeutic target [46]. Here, we show that the effectiveness of MSC-derived EVs against radiation-induced intestinal toxicity could be related to their ability to maintain intestinal barrier integrity.

One of the manifestations of radiation-induced small intestinal toxicity is loss of the self-renewal ability of the epithelium and the subsequent mucosal atrophy associated with dysregulation of the apoptosis/proliferation balance. This can alter the mucosal barrier. In this study, we observed that mucosal atrophy in mice after exposure to 10 Gy WBI was minimized by MSC-derived EVs. An increase in mucosal thickness is probably mediated by the ability of MSC-derived EVs to protect epithelial cells from radiation-induced toxicity. These results are consistent with a previous in vitro finding that irradiation enhances the internalization of MSC-derived exosomes in epithelial cells, leading to greater recipient cell viability [48]. The protection of other epithelial cell types by the administration of MSC-derived exosomes has already been described for cutaneous wound healing [49], kidney toxicity rescue [30], and liver regeneration [31].

Another manifestation of radiation-induced GI toxicity is the enhancement of permeability in the GI mucosa after barrier disruption. A 10-Gy WBI dose led to a rapid and drastic gut permeability increase that, in our experiments, may result from barrier impairment in both small intestinal and colonic mucosa. The colon is a part of the gut that is a primary source of pathogens and toxins, meaning that it is sensitive to stress-induced endotoxemia and bacteremia. Shukla et al. showed that the colonic mucosal barrier is highly sensitive to radiation, more so than the barrier formed by the small intestinal epithelium [46]. After 10 Gy WBI, although a critical epithelial cell mass is preserved in the small intestine, we observed a partial reduction and disruption of claudin-3, a transmembrane protein of tight junctions, potentially reflecting a barrier dysfunction in this part of the gut in our model. MSC-derived EVs prevent the transient increase of radiation-induced gut mucosal permeability probably by maintaining tight junction functionality, as our results show for claudin-3. MSC-derived exosomes also preserved gut barrier integrity in necrotizing enterocolitis model, an effect associated with their ability to reduce the incidence and severity of enterocolitis [33]. Partly, by reducing/preventing the first steps of radiation-induced intestinal barrier damage, MSC-derived EVs might provide therapeutic benefits characterized by their ability to delay radiation-induced death. Our results on crypt cell apoptosis/proliferation and epithelial permeability of the irradiated/EV-treated small intestine show that the preservation of gut barrier integrity is one of the decisive parameters that must be controlled to reduce radiation toxicity.

Nevertheless, the therapeutic benefit of MSC-derived EVs could also be expected to involve partial protection of the HP system. Recent studies have demonstrated that MSC-derived EVs given after lethal WBI enhance long-term survival. This effect is associated with the recovery of HP cells, particularly the white blood cells, which probably occurs through the MSC-derived EVs targeting hematopoietic stem cells [50]. Consistent with these studies, we also observed reduced short-term WBI-induced myelosuppression, evidenced by a reduction in hemorrhaging and the presence of hematopoietic cells in bone marrow slices from MSC-derived EV-treated mice (in supplementary data 3). The potential effects of MSC-derived EVs on radiation-induced toxicity in the HP system should be thoroughly investigated and characterized to optimize treatment for the management of ARS. Further experiments are needed, to establish whether MSC-derived EVs have an effect on other organs or tissues altered by irradiation.

The quality of ARS management also depends on the expediency of the medical support provided to exposed victims. Schoefinius et al. showed that MSC-derived EVs accelerated the kinetics of thrombocyte recovery after lethal WBI compared with MSC themselves [50]. In our study, we also observed that small intestinal mucosa self-renewed more rapidly and with more efficient in mice treated with MSC-derived EVs than in mice treated with MSC. We noted rapid benefits of MSC-derived EVs, as early as 24 h after WBI, which positions MSC-derived EVs as a very good candidate for ARS management as they can act quickly on both the HP and GI systems.

Consequently, we provide proof of concept that MSC-derived EVs are therapeutically beneficial after WBI. Despite the high doses of MSC-derived EVs we used in this study, the treatments could only delay but not prevent death in the WBI animals. So far, most of the treatments that provide promising benefits in management of GI syndrome are administered at very frequent in a long-term iterative process. For instance, BDP/ObreShielf™ (beclomethasone 17,21-dipropionate) provided benefit in radiation-induced GI toxicity when the animals received 2 mg orally every 6 h for 14 days, then 2 mg orally twice daily until 100 days. Consequently, the size and frequency of MSC exosome dose may be important in improving the survival of WBI animals. New investigations to test longer MSC-derived EV therapy are currently ongoing.

## Conclusions

In summary, our results highlight new therapeutic options for patients suffering from ARS. The mechanism of action we propose for the efficacy of MSC-derived EVs in delaying radiation-induced death is consistent with the current consensus that the preservation of gut barrier integrity is key to the management of radiation toxicity. The administration of MSC-derived EVs in relation to the use of MSC themselves may have many advantages. Treatment with MSC-derived EVs is a cell-free therapy and so might overcome the potential side effects associated with native MSC treatment, such as immune reactions, pulmonary occlusion after systemic administration, ectopic tissue genesis, or long-term mal-differentiation of transplanted cells. Moreover, the establishment of EV banks and the possibility of lyophilizing MSC-derived EVs may be valuable in ARS management, especially in the case of large-scale nuclear/radiological events.

## Supplementary information

**Additional file 1: Supplementary data 1.** Characterization of EVs. (a) The size distribution of EVs as measured by Nanoparticle Tracking Analysis on a Zetaview over a size range of 5 to 1000 nm at 25°C. (b) TEM micrograph of EVs immune-labeled with mouse monoclonal anti-CD81 antibody followed by goat anti-mouse secondary antibody coupled with 6 nm gold. The scale bar is 100 nm.

**Additional file 2: Supplementary data 2.** Weight loss following WBI. NUDE mice were subjected to 15 Gy, 13 Gy, 12 Gy, and 10 Gy lethal doses of WBI. Weights of mice in the non-irradiated and WBI groups were measured at 5 days. The results represent a value average of 3 independently repeated experiments (*N*=3, a minimum of 22 animals per group). The results are expressed as percentages of weight loss compared with the control value. **p<0.001, compared with the non-irradiated group and ^$$^p<0.001 compared with the 10Gy WBI group, ^#^*p*<0.05, ^##^p<0.001, compared to the 12 Gy WBI group.

**Additional file 3: Supplementary data 3.** EVs slightly reduce 10Gy WBI-induced histological bone marrow alterations at 3 days. Nude mice received 10Gy WBI. A total of 600 μg EVs was intravenously administered in three 200 μg injections 6, 24 and 48h after WBI. Controls received vehicle. Femur samples were incubated for 4 hours in decalcification buffer (#320715-1000, Ral Diagnostic) before inclusion in paraffin. Formalin-fixed, paraffin-embedded femur was cut into 5 μm sectionson a rotary microtome (Leica Microsystems AG, Wetzlar, Germany) and mounted on polylysine slides. The sections were deparaffinized in xylene and rehydrated through ethanol baths and PBS. The Hydrated sections were stained with haematoxylin, eosin, and Safran (HES). Representative pictures of femoral bone marrow slice observed 3 days after WBI in (a) non-irradiated NUDE mice, (b) NUDE mice subjected to 10Gy WBI and (c) NUDE mice subjected to 10Gy WBI and treated with MSC-derived EVs. Scale bar 100μm for (a), (b), (c) and 50μm for (d).

**Additional file 4: Supplementary data 4.** Allogenic total bone marrow transplantation does not prevent WBI-induced NUDE mice death. Nude mice received 15, 13 or 11 Gy WBI. For each dose of WBI, mice were randomly assigned to two different groups. Control mice received the vehicle (first group). Two hours after WBI, a single allogenic transplantation of 5 millions total BM cells was performed intravenously by retro-orbital injection (second group). (a) In a first batch of the experiment, femur samples were taken 7 days after 15 Gy WBI and then incubated for 4 hours in decalcification buffer (#320715-1000, Ral Diagnostic) before inclusion in paraffin. Formalin-fixed, paraffin-embedded femur was cut into 5 μm sections on a rotary microtome (Leica Microsystems AG, Wetzlar, Germany) and mounted on polylysine slides. The sections were deparaffinized in xylene and rehydrated through ethanol baths and PBS. The Hydrated sections were stained with haematoxylin, eosin, and Safran (HES). Representative pictures of femoral bone marrow slice observed 7 days after WBI in (aa) non-irradiated NUDE mice (4 animals), (ab) NUDE mice subjected to 15Gy WBI and treated with PBS (3 animals) and (ac) NUDE mice subjected to 15Gy WBI and treated with total BM cells (3 animals). Scale bar 50μm. (b,c,d) In a second batch of the experiment, the survival rate of mice was evaluated after 15 Gy (b), 13 Gy (c) or 11 Gy (d) with or without total BM transplantation. Each value results from one representative experiment (*N*=1, 9 mice in the 11 Gy WBI group treated with PBS, 9 mice in the 11 Gy WBI group treated with BM, 10 mice in the 13 Gy WBI group treated with PBS, 10 mice in the 13 Gy WBI group treated with BM, 10 mice in the 15 Gy WBI group treated with PBS, and 10 mice in the 15 Gy WBI group treated with BM). The mouse survival curves were calculated using Kaplan–Meier method and the *P*-value was determined by the log-rank test eventually adjusted for Multiple Comparisons. No significant differences were reported.

**Additional file 5: Supplementary data 5.** Severity of histological alterations of the small intestine is WBI dose-dependent. NUDE mice were subjected to 15 Gy, 13 Gy, 12 Gy, and 10 Gy lethal doses of WBI. Three days after WBI, small intestinal sections were stained with Haematoxylin-Eosin-Safran (HES). Morphometric analysis was performed: quantitative assessment of (a) crypt depth (μm) and (b) small intestinal crypt/villus density (number of crypt/mm of the small intestine). Each value represents the average of 30-100 independent measurements per animal (where the decreasing doses we observed on each section increased the number of measurable crypts and villi) coming from 2 independently repeated experiments (*N*=2, 3 to 6 animals per group). *p≤0.05, **p≤0.001 compared with the non-irradiated group and ^$^*p*<0.05 compared with the 10Gy WBI group.

**Additional file 6 : Supplementary data 6:** EV ability to slightly protect the small intestine from radiation-induced villus height reduction is dose-dependent. Nude mice received 10 Gy WBI. A total of 600, 300 or 150 μg of MSC-derived EVs was intravenously administered in three injections of 300, 200, 100 and 50 μg respectively, 6h, 24h and 48h after WBI. Regarding ethical considerations concerning intravenously volume administration in mice, we were not able to treat animals with higher concentration of MSC-derived EVs. Controls received the vehicle. Three days after WBI, small intestinal sections were stained with Haematoxylin-Eosin-Safran (HES). Quantitative analysis of villus height of the small intestine (μm) in non-irradiated NUDE mice, NUDE mice subjected to WBI and NUDE mice subjected to WBI and treated with MSC-derived EVs. Each value represents the average of 20-30 independent measurements per animal (*N*=1, 4 to 8 animals per group). **p<0.001 compared with the the control group.

## Data Availability

The datasets generated and analyzed during the current study are available.

## Abbreviations

ARS: Acute radiation syndrome
EVs: Extracellular vehicles
GI: Gastrointestinal
Gy: Grays
HP: Hematopoietic
MSC: Mesenchymal stromal cells
WBI: Whole-body irradiation

## References

[1] Allman T (2013). radiation sickness: Thomson Reuters.

[2] Macia IGM, Lucas Calduch A, Lopez EC (2011). Radiobiology of the acute radiation syndrome. Rep Pract Oncol Radiother.

[3] Donnelly EH, Nemhauser JB, Smith JM, Kazzi ZN, Farfan EB, Chang AS (2010). Acute radiation syndrome: assessment and management. South Med J.

[4] Douglass M (2018). Eric J. Hall and Amato J. Giaccia: Radiobiology for the radiologist. Australas Phys Eng Sci Med.

[5] Pacelli R, Mansi L. Eric Hall and Amato J. Giaccia: Radiobiology for the radiologist, 6th edn. Eur J Nucl Med Mol Imaging 2007;34(6):965–966.

[6] Williams JP, McBride WH (2011). After the bomb drops: a new look at radiation-induced multiple organ dysfunction syndrome (MODS). Int J Radiat Biol.

[7] Fliedner TM, Dörr HD, Meineke V (2005). Multi-organ involvement as a pathogenetic principle of the radiation syndromes: a study involving 110 case histories documented in SEARCH and classified as the bases of haematopoietic indicators of effect. Br J Radiol.

[8] Waselenko JK, MacVittie TJ, Blakely WF, Pesik N, Wiley AL, Dickerson WE (2004). Medical management of the acute radiation syndrome: recommendations of the Strategic National Stockpile Radiation Working Group. Ann Intern Med.

[9] Guinan EC, Barbon CM, Kalish LA, Parmar K, Kutok J, Mancuso CJ (2011). Bactericidal/permeability-increasing protein (rBPI21) and fluoroquinolone mitigate radiation-induced bone marrow aplasia and death. Sci Transl Med.

[10] Potten CS (2004). Radiation, the ideal cytotoxic agent for studying the cell biology of tissues such as the small intestine. Radiat Res.

[11] Gorin NC, Fliedner TM, Gourmelon P, Ganser A, Meineke V, Sirohi B (2006). Consensus conference on European preparedness for haematological and other medical management of mass radiation accidents. Ann Hematol.

[12] Fliedner TM, Chao NJ, Bader JL, Boettger A, Case C, Chute J (2009). Stem cells, multiorgan failure in radiation emergency medical preparedness: a U.S./European Consultation Workshop. Stem Cells.

[13] Mason KA, Withers HR, McBride WH, Davis CA, Smathers JB (1989). Comparison of the gastrointestinal syndrome after total-body or total-abdominal irradiation. Radiat Res.

[14] Terry NH, Travis EL (1989). The influence of bone marrow depletion on intestinal radiation damage. Int J Radiat Oncol Biol Phys.

[15] Eaton EB, Varney TR (2015). Mesenchymal stem cell therapy for acute radiation syndrome: innovative medical approaches in military medicine. Mil Med Res..

[16] Fukumoto R (2016). Mesenchymal stem cell therapy for acute radiation syndrome. Mil Med Res.

[17] Caplan AI, Dennis JE (2006). Mesenchymal stem cells as trophic mediators. J Cell Biochem.

[18] Semont A, Demarquay C, Bessout R, Durand C, Benderitter M, Mathieu N (2013). Mesenchymal stem cell therapy stimulates endogenous host progenitor cells to improve colonic epithelial regeneration. PLoS One.

[19] Lai RC, Arslan F, Lee MM, Sze NS, Choo A, Chen TS (2010). Exosome secreted by MSC reduces myocardial ischemia/reperfusion injury. Stem Cell Res.

[20] Biancone L, Bruno S, Deregibus MC, Tetta C, Camussi G (2012). Therapeutic potential of mesenchymal stem cell-derived microvesicles. Nephrol Dial Transplant.

[21] Witwer KW, Van Balkom BWM, Bruno S, Choo A, Dominici M, Gimona M (2019). Defining mesenchymal stromal cell (MSC)-derived small extracellular vesicles for therapeutic applications. J Extracell Vesicles..

[22] Harding C, Heuser J, Stahl P (1983). Receptor-mediated endocytosis of transferrin and recycling of the transferrin receptor in rat reticulocytes. J Cell Biol.

[23] Pan BT, Johnstone RM (1983). Fate of the transferrin receptor during maturation of sheep reticulocytes in vitro: selective externalization of the receptor. Cell..

[24] Cocucci E, Meldolesi J (2015). Ectosomes and exosomes: shedding the confusion between extracellular vesicles. Trends Cell Biol.

[25] Raposo G, Stoorvogel W (2013). Extracellular vesicles: exosomes, microvesicles, and friends. J Cell Biol.

[26] Tan SS, Yin Y, Lee T, Lai RC, Yeo RW, Zhang B, et al. Therapeutic MSC exosomes are derived from lipid raft microdomains in the plasma membrane. J Extracell Vesicles. 2013:1–10. 10.3402/jev.v2i0.22614 PMC3873122 24371518

[27] Lai RC, Tan SS, Yeo RW, Choo AB, Reiner AT, Su Y (2016). MSC secretes at least 3 EV types each with an unique permutation of membrane lipid, protein and RNA. J Extracell Vesicles.

[28] Thery C, Witwer KW, Aikawa E, Alcaraz MJ, Anderson JD, Andriantsitohaina R (2018). Minimal information for studies of extracellular vesicles 2018 (MISEV2018): a position statement of the International Society for Extracellular Vesicles and update of the MISEV2014 guidelines. J Extracell Vesicles..

[29] Phinney DG (2017). Mesenchymal stromal cells and ischemic heart disease: hitting the target?. Cardiovasc Diagn Ther.

[30] Zhou Y, Xu H, Xu W, Wang B, Wu H, Tao Y (2013). Exosomes released by human umbilical cord mesenchymal stem cells protect against cisplatin-induced renal oxidative stress and apoptosis in vivo and in vitro. Stem Cell Res Ther.

[31] Tan CY, Lai RC, Wong W, Dan YY, Lim SK, Ho HK (2014). Mesenchymal stem cell-derived exosomes promote hepatic regeneration in drug-induced liver injury models. Stem Cell Res Ther.

[32] Zhang B, Wang M, Gong A, Zhang X, Wu X, Zhu Y (2015). HucMSC-exosome mediated-Wnt4 signaling is required for cutaneous wound healing. Stem Cells.

[33] Rager TM, Olson JK, Zhou Y, Wang Y, Besner GE (2016). Exosomes secreted from bone marrow-derived mesenchymal stem cells protect the intestines from experimental necrotizing enterocolitis. J Pediatr Surg.

[34] Kordelas L, Rebmann V, Ludwig AK, Radtke S, Ruesing J, Doeppner TR (2014). MSC-derived exosomes: a novel tool to treat therapy-refractory graft-versus-host disease. Leukemia..

[35] Zhang B, Yin Y, Lai RC, Tan SS, Choo AB, Lim SK (2014). Mesenchymal stem cells secrete immunologically active exosomes. Stem Cells Dev.

[36] Alcayaga-Miranda F, Gonzalez PL, Lopez-Verrilli A, Varas-Godoy M, Aguila-Diaz C, Contreras L (2016). Prostate tumor-induced angiogenesis is blocked by exosomes derived from menstrual stem cells through the inhibition of reactive oxygen species. Oncotarget..

[37] Chen W, Huang Y, Han J, Yu L, Li Y, Lu Z (2016). Immunomodulatory effects of mesenchymal stromal cells-derived exosome. Immunol Res.

[38] Fouillard L, Bensidhoum M, Bories D, Bonte H, Lopez M, Moseley AM (2003). Engraftment of allogeneic mesenchymal stem cells in the bone marrow of a patient with severe idiopathic aplastic anemia improves stroma. Leukemia..

[39] Semont A, Mouiseddine M, Francois A, Demarquay C, Mathieu N, Chapel A (2010). Mesenchymal stem cells improve small intestinal integrity through regulation of endogenous epithelial cell homeostasis. Cell Death Differ.

[40] Chen TS, Arslan F, Yin Y, Tan SS, Lai RC, Choo AB (2011). Enabling a robust scalable manufacturing process for therapeutic exosomes through oncogenic immortalization of human ESC-derived MSCs. J Transl Med.

[41] Sze SK, de Kleijn DP, Lai RC, Khia way Tan E, Zhao H, Yeo KS (2007). Elucidating the secretion proteome of human embryonic stem cell-derived mesenchymal stem cells. Mol Cell Proteomics.

[42] Tambuwala MM, Cummins EP, Lenihan CR, Kiss J, Stauch M, Scholz CC (2010). Loss of prolyl hydroxylase-1 protects against colitis through reduced epithelial cell apoptosis and increased barrier function. Gastroenterology..

[43] Cox C (1988). Multinomial regression models based on continuation ratios. Stat Med.

[44] Paris F, Fuks Z, Kang A, Capodieci P, Juan G, Ehleiter D (2001). Endothelial apoptosis as the primary lesion initiating intestinal radiation damage in mice. Science..

[45] Konig J, Wells J, Cani PD, Garcia-Rodenas CL, MacDonald T, Mercenier A (2016). Human intestinal barrier function in health and disease. Clin Transl Gastroenterol.

[46] Shukla PK, Gangwar R, Manda B, Meena AS, Yadav N, Szabo E (2016). Rapid disruption of intestinal epithelial tight junction and barrier dysfunction by ionizing radiation in mouse colon in vivo: protection by N-acetyl-l-cysteine. Am J Physiol Gastrointest Liver Physiol.

[47] Miyoshi Y, Tanabe S, Suzuki T (2016). Cellular zinc is required for intestinal epithelial barrier maintenance via the regulation of claudin-3 and occludin expression. Am J Physiol Gastrointest Liver Physiol.

[48] Hazawa M, Tomiyama K, Saotome-Nakamura A, Obara C, Yasuda T, Gotoh T (2014). Radiation increases the cellular uptake of exosomes through CD29/CD81 complex formation. Biochem Biophys Res Commun.

[49] Zhang J, Guan J, Niu X, Hu G, Guo S, Li Q (2015). Exosomes released from human induced pluripotent stem cells-derived MSCs facilitate cutaneous wound healing by promoting collagen synthesis and angiogenesis. J Transl Med.

[50] Schoefinius JS, Brunswig-Spickenheier B, Speiseder T, Krebs S, Just U, Lange C (2017). Mesenchymal stromal cell-derived extracellular vesicles provide long-term survival after total body irradiation without additional hematopoietic stem cell support. Stem Cells.

