# Supplementary figures and images for: Extracellular vesicles derived from mesenchymal stromal cells mitigate intestinal toxicity in a mouse model of acute radiation syndrome
Source: Stem Cell Res Ther. 2020 Aug 27;11:371. doi: 10.1186/s13287-020-01887-1 (PMC7457304; doi:10.1186/s13287-020-01887-1)

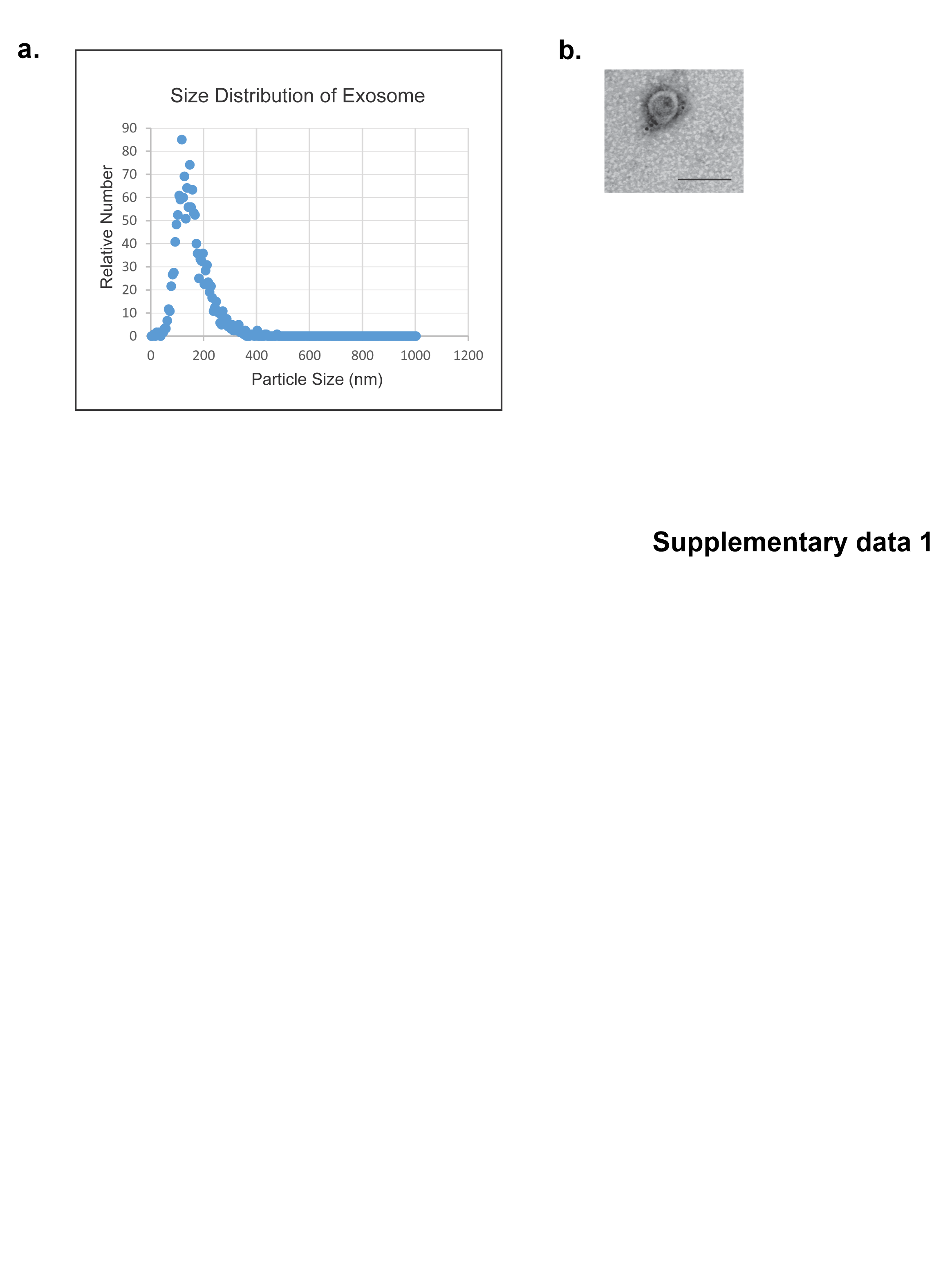

Supplement: Supplementary file 1 — Additional file 1: Supplementary data 1. Characterization of EVs. (a) The size distribution of EVs as measured by Nanoparticle Tracking Analysis on a Zetaview over a size range of 5 to 1000 nm at 25°C. (b) TEM micrograph of EVs immune-labeled with mouse monoclonal anti-CD81 antibody followed by goat anti-mouse secondary antibody coupled with 6 nm gold. The scale bar is 100 nm. [file 13287_2020_1887_MOESM1_ESM.tif]

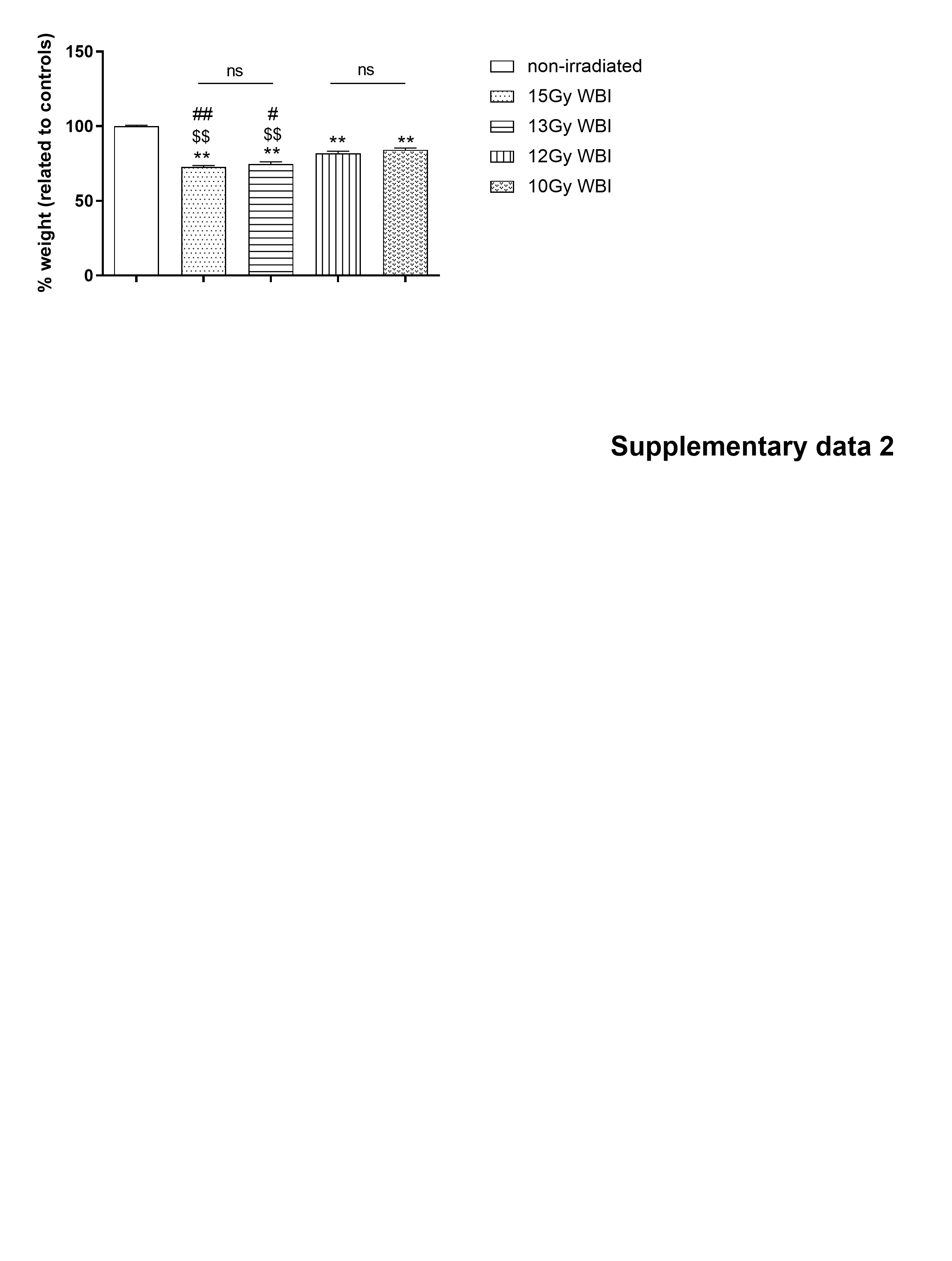

Supplement: Supplementary file 2 — Additional file 2: Supplementary data 2. Weight loss following WBI. NUDE mice were subjected to 15 Gy, 13 Gy, 12 Gy, and 10 Gy lethal doses of WBI. Weights of mice in the non-irradiated and WBI groups were measured at 5 days. The results represent a value average of 3 independently repeated experiments (N=3, a minimum of 22 animals per group). The results are expressed as percentages of weight loss compared with the control value. **p<0.001, compared with the non-irradiated group and $$p<0.001 compared with the 10Gy WBI group, #p<0.05, ##p<0.001, compared to the 12 Gy WBI group. [file 13287_2020_1887_MOESM2_ESM.tif]

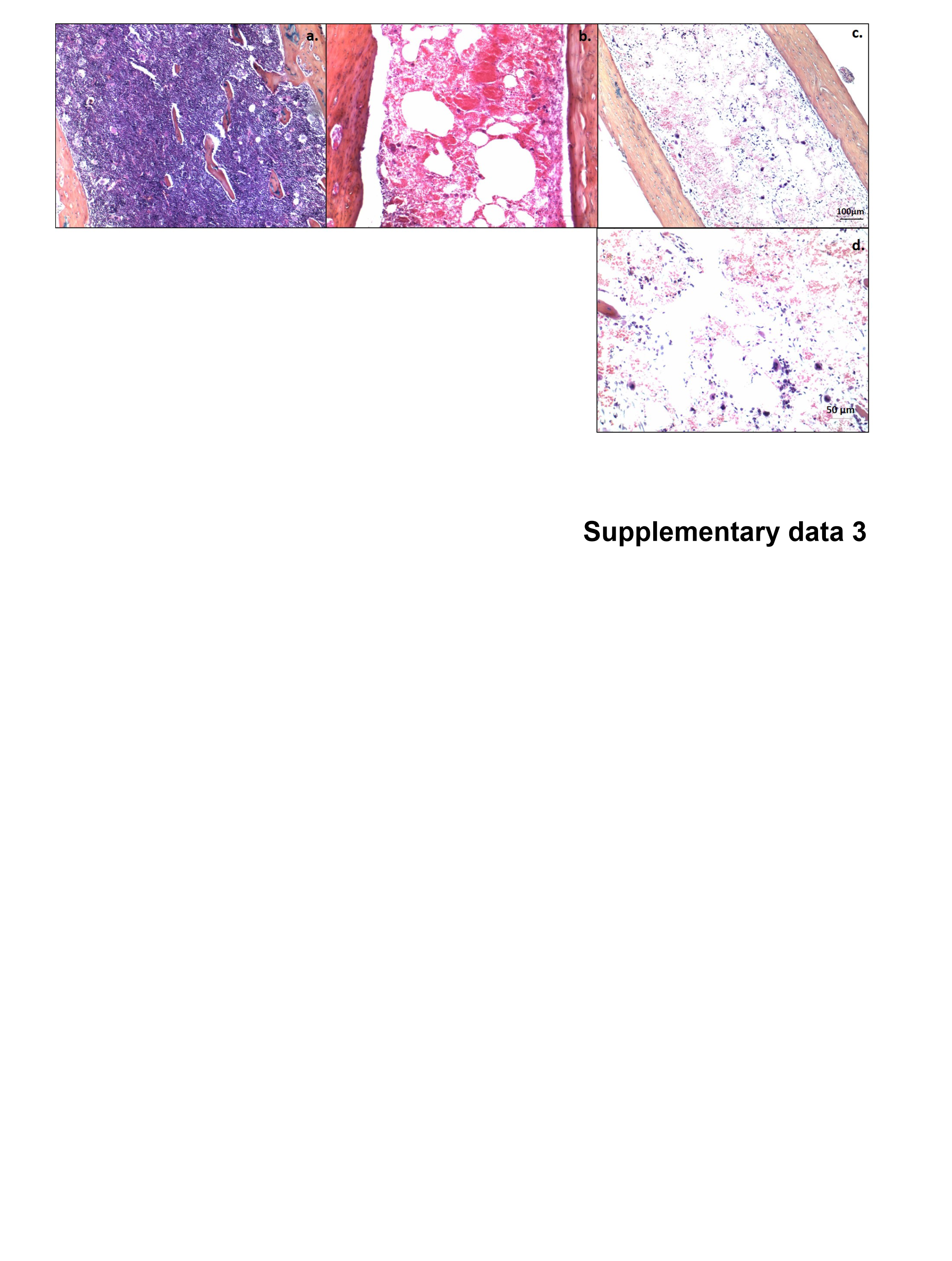

Supplement: Supplementary file 3 — Additional file 3: Supplementary data 3. EVs slightly reduce 10Gy WBI-induced histological bone marrow alterations at 3 days. Nude mice received 10Gy WBI. A total of 600 μg EVs was intravenously administered in three 200 μg injections 6, 24 and 48h after WBI. Controls received vehicle. Femur samples were incubated for 4 hours in decalcification buffer (#320715-1000, Ral Diagnostic) before inclusion in paraffin. Formalin-fixed, paraffin-embedded femur was cut into 5 μm sectionson a rotary microtome (Leica Microsystems AG, Wetzlar, Germany) and mounted on polylysine slides. The sections were deparaffinized in xylene and rehydrated through ethanol baths and PBS. The Hydrated sections were stained with haematoxylin, eosin, and Safran (HES). Representative pictures of femoral bone marrow slice observed 3 days after WBI in (a) non-irradiated NUDE mice, (b) NUDE mice subjected to 10Gy WBI and (c) NUDE mice subjected to 10Gy WBI and treated with MSC-derived EVs. Scale bar 100μm for (a), (b), (c) and 50μm for (d). [file 13287_2020_1887_MOESM3_ESM.tif]

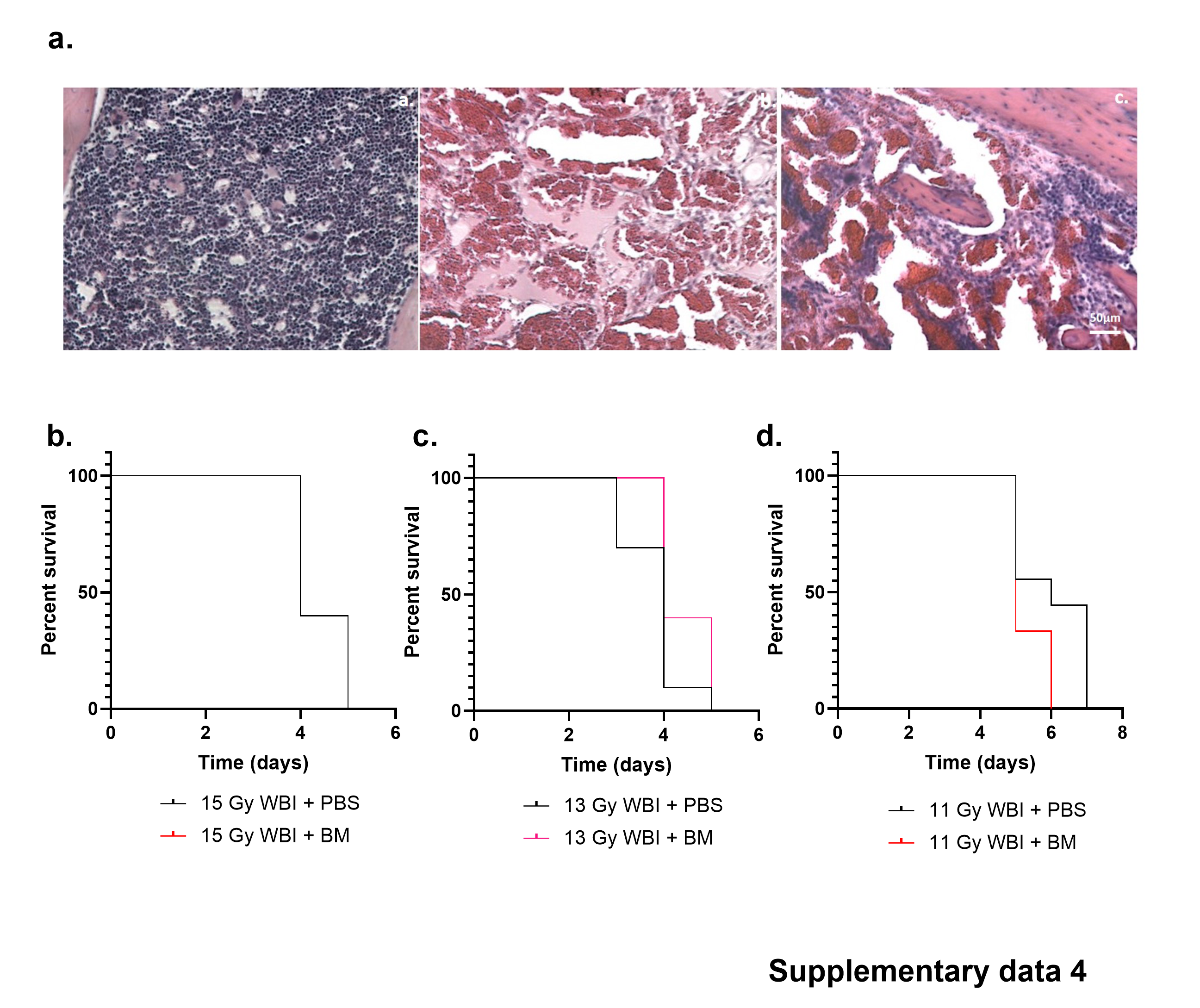

Supplement: Supplementary file 4 — Additional file 4: Supplementary data 4. Allogenic total bone marrow transplantation does not prevent WBI-induced NUDE mice death. Nude mice received 15, 13 or 11 Gy WBI. For each dose of WBI, mice were randomly assigned to two different groups. Control mice received the vehicle (first group). Two hours after WBI, a single allogenic transplantation of 5 millions total BM cells was performed intravenously by retro-orbital injection (second group). (a) In a first batch of the experiment, femur samples were taken 7 days after 15 Gy WBI and then incubated for 4 hours in decalcification buffer (#320715-1000, Ral Diagnostic) before inclusion in paraffin. Formalin-fixed, paraffin-embedded femur was cut into 5 μm sections on a rotary microtome (Leica Microsystems AG, Wetzlar, Germany) and mounted on polylysine slides. The sections were deparaffinized in xylene and rehydrated through ethanol baths and PBS. The Hydrated sections were stained with haematoxylin, eosin, and Safran (HES). Representative pictures of femoral bone marrow slice observed 7 days after WBI in (aa) non-irradiated NUDE mice (4 animals), (ab) NUDE mice subjected to 15Gy WBI and treated with PBS (3 animals) and (ac) NUDE mice subjected to 15Gy WBI and treated with total BM cells (3 animals). Scale bar 50μm. (b,c,d) In a second batch of the experiment, the survival rate of mice was evaluated after 15 Gy (b), 13 Gy (c) or 11 Gy (d) with or without total BM transplantation. Each value results from one representative experiment (N=1, 9 mice in the 11 Gy WBI group treated with PBS, 9 mice in the 11 Gy WBI group treated with BM, 10 mice in the 13 Gy WBI group treated with PBS, 10 mice in the 13 Gy WBI group treated with BM, 10 mice in the 15 Gy WBI group treated with PBS, and 10 mice in the 15 Gy WBI group treated with BM). The mouse survival curves were calculated using Kaplan–Meier method and the P-value was determined by the log-rank test eventually adjusted for Multiple Comparisons. No significant differences [file 13287_2020_1887_MOESM4_ESM.tif]

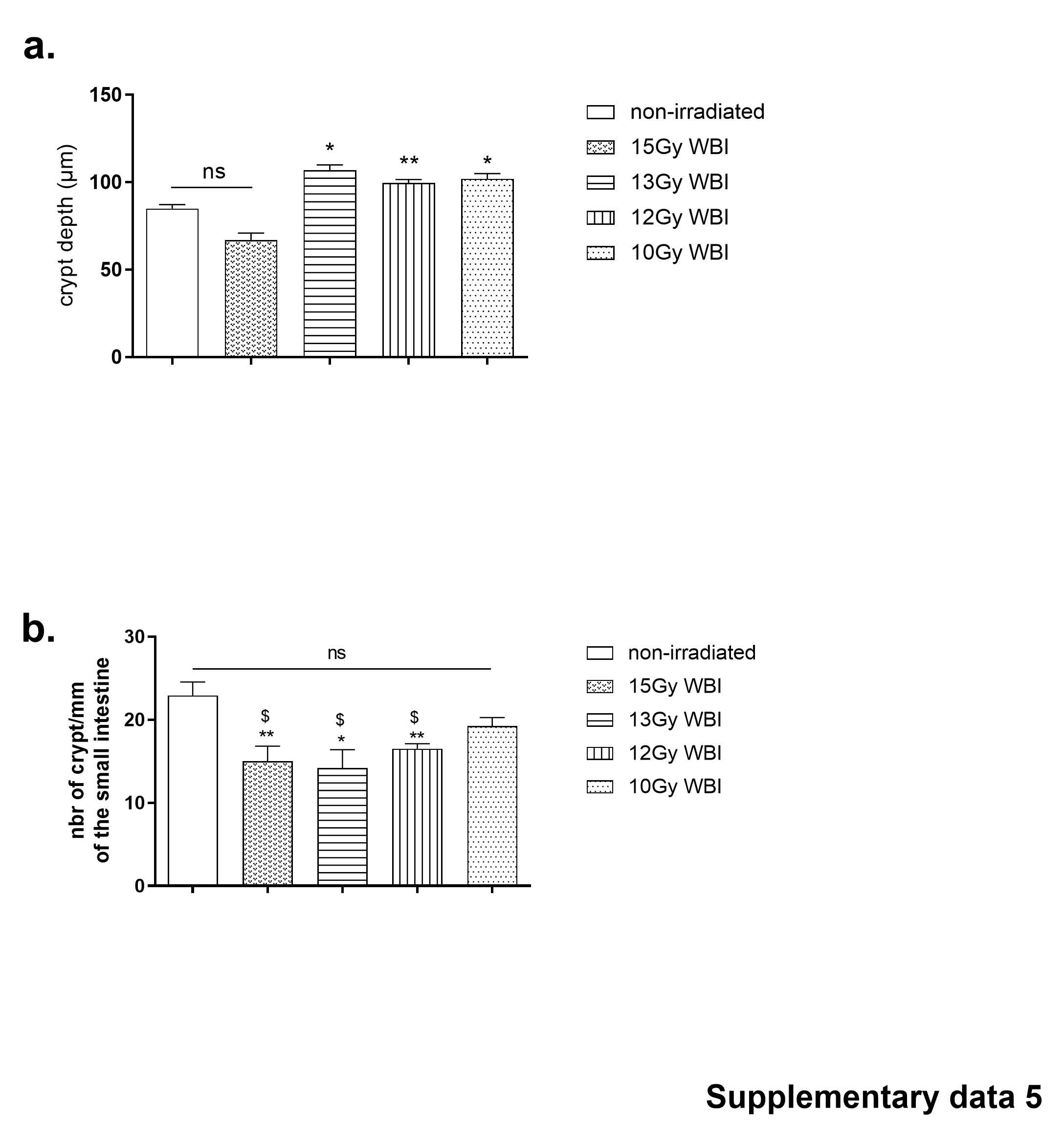

Supplement: Supplementary file 5 — Additional file 5: Supplementary data 5. Severity of histological alterations of the small intestine is WBI dose-dependent. NUDE mice were subjected to 15 Gy, 13 Gy, 12 Gy, and 10 Gy lethal doses of WBI. Three days after WBI, small intestinal sections were stained with Haematoxylin-Eosin-Safran (HES). Morphometric analysis was performed: quantitative assessment of (a) crypt depth (μm) and (b) small intestinal crypt/villus density (number of crypt/mm of the small intestine). Each value represents the average of 30-100 independent measurements per animal (where the decreasing doses we observed on each section increased the number of measurable crypts and villi) coming from 2 independently repeated experiments (N=2, 3 to 6 animals per group). *p≤0.05, **p≤0.001 compared with the non-irradiated group and $p<0.05 compared with the 10Gy WBI group. [file 13287_2020_1887_MOESM5_ESM.tif]

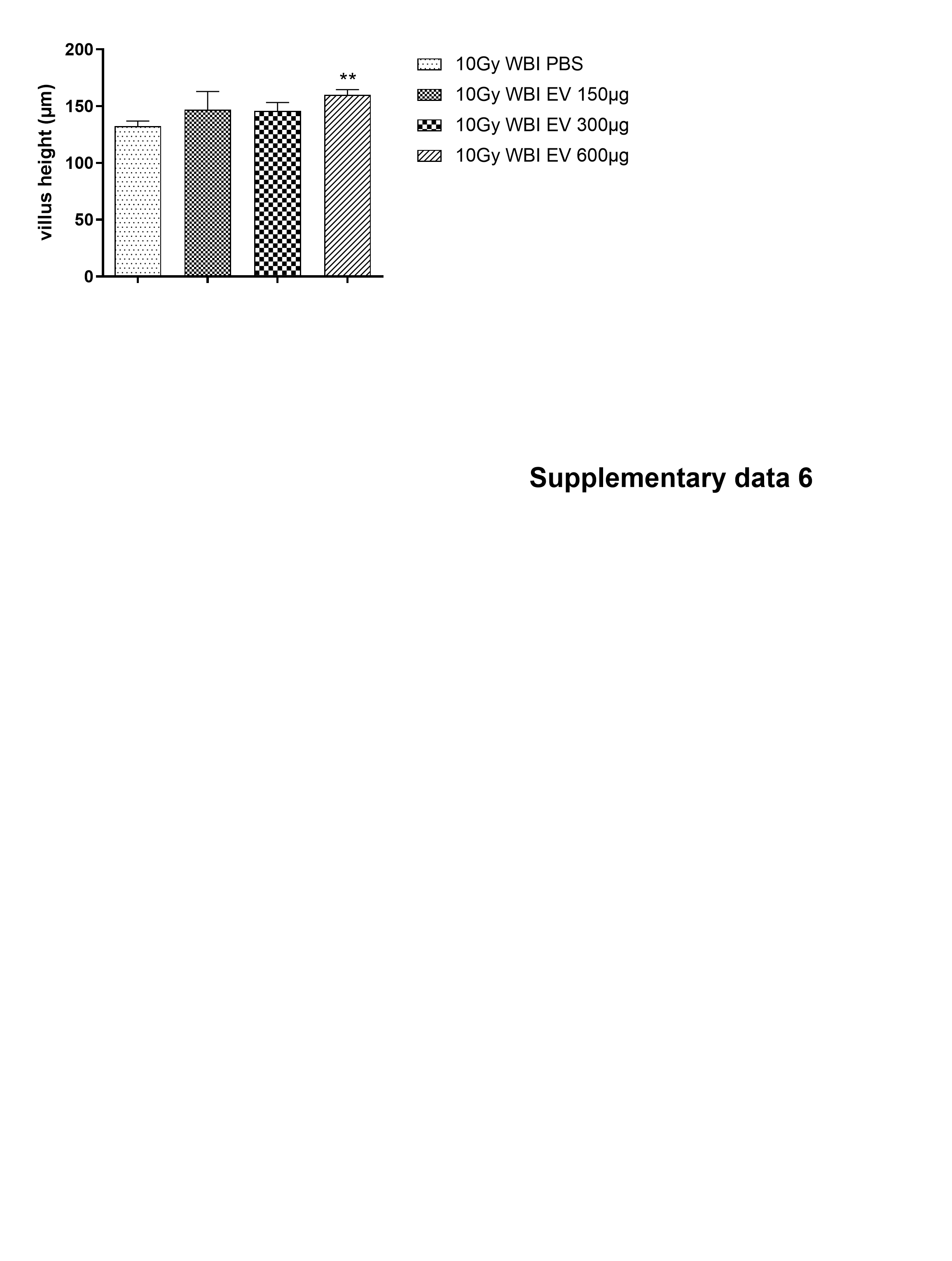

Supplement: Supplementary file 6 — Additional file 6 : Supplementary data 6: EV ability to slightly protect the small intestine from radiation-induced villus height reduction is dose-dependent. Nude mice received 10 Gy WBI. A total of 600, 300 or 150 μg of MSC-derived EVs was intravenously administered in three injections of 300, 200, 100 and 50 μg respectively, 6h, 24h and 48h after WBI. Regarding ethical considerations concerning intravenously volume administration in mice, we were not able to treat animals with higher concentration of MSC-derived EVs. Controls received the vehicle. Three days after WBI, small intestinal sections were stained with Haematoxylin-Eosin-Safran (HES). Quantitative analysis of villus height of the small intestine (μm) in non-irradiated NUDE mice, NUDE mice subjected to WBI and NUDE mice subjected to WBI and treated with MSC-derived EVs. Each value represents the average of 20-30 independent measurements per animal (N=1, 4 to 8 animals per group). **p<0.001 compared with the the control group. [file 13287_2020_1887_MOESM6_ESM.tif]
